# Intelligent Rock‐Climbing Robot Capable of Multimodal Locomotion and Hybrid Bioinspired Attachment

**DOI:** 10.1002/advs.202309058

**Published:** 2024-07-15

**Authors:** Peijin Zi, Kun Xu, Jiawei Chen, Chang Wang, Tao Zhang, Yang Luo, Yaobin Tian, Li Wen, Xilun Ding

**Affiliations:** ^1^ School of Mechanical Engineering and Automation Beihang University Beijing 100191 China; ^2^ School of Electro‐mechanical Engineering Guangdong University of Technology Guangzhou 510006 China

**Keywords:** bioinspired adhesion, bioinspired robots, biomimetics, climbing robots, robotics

## Abstract

Rock‐climbing robots have significant potential in fieldwork and planetary exploration. However, they currently face limitations such as a lack of stability and adaptability on extreme terrains, slow locomotion, and single functionality. This study introduces a novel multimodal and adaptive rock‐climbing robot (MARCBot), which addresses these limitations through spiny grippers that draw inspiration from morpho‐functionalities observed in beetles, arboreal birds, and hoofed animals. This hybrid bioinspired design enables high attachment strength, passive adaptability to different terrains, and quick attachment on rock surfaces. The multimodal functionality of the gripper allows for attachment during climbing and support during walking. A novel control strategy using dynamics and quadratic programming (QP) optimizes attachment wrench distribution, reducing cost‐of‐transport by 20.03% and 6.05% compared to closed‐loop inverse kinematic (CLIK) and virtual model control (VMC) methods, respectively. MARCBot achieved climbing speeds of 0.15 m min^−1^ on a vertical discrete rock surface under gravity and trotting speeds of up to 0.21 m s^−1^ on various complex terrains. It is the first robot capable of climbing on rock surfaces and trotting in complex terrains without the need for switching end‐effectors. This study highlights significant advancements in climbing and multimodal locomotion for robots in extreme environments.

## Graphical abstract

This study presents MARCBot, a quadruped robot capable of rock climbing and fast trotting without the need to switch end effectors. With its hybrid bioinspired spiny gripper and quasi‐whole‐body control, MARCBot can navigate challenging rocky terrains and traverse diverse complex terrains. MARCBot is the first of its kind to achieve such capabilities, making it a significant contribution to the field.

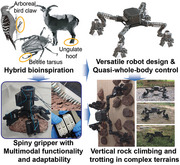

## 1 Introduction

Climbing robots are a type of service and mobile robot that primarily operate on inclined or vertical surfaces and hold great promise for applications such as construction cleaning, field rescue, and asteroid and planetary exploration.^[^ ^1^ ^]^ Technological advancement has allowed humans to explore the surfaces of more extra‐terrestrial bodies, such as the Moon, Mars, and asteroids. In these celestial bodies, nonstructural environments, such as steep slopes and rugged rocks, require rock‐climbing robots capable of reliable attachment in complex environments.^[^ ^2^ ^]^ Such robots are crucial for future exploration and sampling of extraterrestrial planets and asteroids.^[^ ^3^ ^]^ They can also be used for scouting and rescue at disaster sites or in hazardous areas, deep‐ocean exploration, and geological surveys.^[^ ^4^ ^]^ The key technologies of such robots include attachment technology, robot mechanical design, and control methods.

Conventional adhesion methods (e.g., negative pressure,^[^ ^5^ ^]^ pneumatic force,^[^ ^6^ ^]^ electrostatic adhesion,^[^ ^7^ ^]^ and magnetic adhesion^[^ ^8^ ^]^) have been successfully used for robotic climbing and perching. However, climbing robots that use these methods struggle to adapt to complex and nonstructural natural environments. The high climbing ability and attachment mechanism exhibited by animals during foraging and survival have served as the foundation to be imitated for the design of climbing robots.^[^ ^1^, ^9^ ^]^ Progress has been made in the study of animal scaling mechanisms on different surfaces, such as dry adhesion of geckos,^[^ ^10^ ^]^ wet adhesion of tree frogs,^[^ ^11^ ^]^ negative pressure adsorption of biological suckers,^[^ ^12^ ^]^ and mechanical interlocking of spines and claws.^[^ ^13^ ^]^ Mechanical adhesion based on claw and spine locking is particularly suitable for attachment to rough surfaces.^[^ ^14^ ^]^ Several types of spine mechanisms have been successfully used in climbing robots. Asdeck et al.^[^ ^14a^ ^]^ used shape deposition manufacturing to create a rigid‐flexible coupled micro spine and a planar attachment model of the mechanical adhesion of the spine. Climbing robots with biomimetic micro spine arrays, such as Spinybot II,^[^ ^14a^ ^]^ RiSE,^[^ ^15^ ^]^ inchworm‐like robot,^[^ ^16^ ^]^ CLIBO,^[^ ^17^ ^]^ DynoClimber,^[^ ^18^ ^]^ BOB 2.0,^[^ ^19^ ^]^ as well as other robots,^[^ ^20^ ^]^ can scale vertical rough walls. These attachment systems consume minimal energy, and power loss may not directly result in system failure.^[^ ^9c^ ^]^ However, microspine arrays cannot produce force closure, resulting in weak mechanical interlocking stability.^[^ ^14a^ ^]^

Many spiny grippers have been developed for stable attachment to nonstructural rock surface environments.^[^ ^21^ ^]^ However, only a few have been integrated into climbing robots. The Jet Propulsion Laboratory has developed a series of climbing robots referred to as LEMUR.^[^ ^22^ ^]^ LEMUR 3^[^ ^23^ ^]^ can climb rock surfaces using spiny grippers with spiny mechanisms similar to those of RiSE. However, because of the complexity of the attachment device (which includes four drive motors), the attachment and detachment processes require ≈3 min. Tohoku University developed a free‐climbing robot^[^ ^24^ ^]^ and Hubrobo.^[^ ^25^ ^]^ The six‐finger passive gripper of Hubrobo is based on a biological finger tendon‐locking mechanism that converts outward pulling forces into energy‐free gripping forces. However, passively actuated grippers are difficult to adjust and exhibit lower attachment forces. They are also unable to climb in a gravity environment. The University of California in Los Angeles has developed a four‐limb climbing robot, SCALER, whose attachment device is a spiny gripper with two underactuated fingers,^[^ ^26^ ^]^ which may be unable to generate a sufficiently large component of the attachment wrench in some directions. The attachment devices of these robots can only perform grasping functionality while climbing and cannot enable the robots to move quickly on the ground.

Overall, the current applications of bioinspired attachment in such robots demonstrate a method of utilizing **mechanical intelligence** to enhance attachment capability and surface adaptability. Mechanical intelligence is a bioinspired concept for transforming engineering designs. It explores nature‐inspired mechanisms for automatic adaptability and translates them into a new generation of mechanically intelligent devices with superior capabilities.^[^ ^27^ ^]^ However, the attachment capability and application scenarios of such robots are limited because of the single morpho‐functionality of their attachment devices. Consequently, current rock‐climbing robots hardly achieve multiterrain adaptability and multimodal locomotion of quickly walking and steadily climbing. **Hybrid bioinspiration** can be used to solve this issue. It can be understood as the integration of different biomimetic design methods and principles to create more efficient and innovative solutions. This approach can involve combining mechanisms and morpho‐functionalities from different organisms or applying biological principles to engineering design to achieve better designs and innovations. By incorporating mechanical intelligence from the attachment and locomotion mechanisms of various animals, along with implementing morphing functionality, it becomes possible to develop multimodal climbing devices with strong adaptability and attachment capabilities.

For the mechanical design, LEMUR 2B has only three degrees of freedom (DOF) in each leg and can move with a specific gait on rock surfaces in a particular configuration. LEMUR 3 has seven DOFs on a single leg.^[^ ^23^ ^]^ Ideally, it should move in any direction on rock surfaces. However, increasing the number of actuators increases their inertia and mass, thereby limiting their dynamic performance and application scenarios. Consequently, it cannot climb rocks under the effect of gravity. Hubrobo^[^ ^25^ ^]^ has three active DOFs and three passive DOFs in one leg for lightweight and dexterity. Nevertheless, a passive ankle may introduce uncertainty in the movement. The leg configuration of SCALER is integrated using a 3‐DOF parallel mechanism to achieve the desired foot position and a 3‐DOF wrist to achieve the desired gripper posture.^[^ ^26a,b^ ^]^ The parallel mechanism enhances the stiffness and load‐bearing capacity of the limbs. However, this design increases the distance from the robot's body and center of mass to the attached substrate, thereby increasing the turnover moment. Few research has been conducted on the DOFs and joint configurations required for climbing robots. The redundancy of DOFs can improve the agility of the robot; however, it also increases the weight and creates design problems in terms of balancing the effective workspace and joint arrangement. Insufficient DOFs can lead to problems such as uncontrollable robot motion and excessive constraint force on the robot limbs.

Another key technology for climbing robots is the control method.^[^ ^1^ ^]^ Position control based on is widely used in multilegged climbing robots.^[^ ^15^, ^17^, ^25^, ^28^ ^]^ This is mainly because their speed and dynamic performance are relatively low, resulting in a low demand for torque control. However, climbing robots may lack the ability to adjust the required attachment force of each foot owing to a lack of joint torque regulation capability. Because a robot is frequently overconstrained during climbing, position errors can generate large internal forces inside the robot, leading to increased energy consumption and required adhesion. If the attachment forces are not optimized and uncontrollable, the attachment devices may overload, posing slip and turnover risks. VMC is a sophisticated control strategy widely used in legged systems.^[^ ^29^ ^]^ It also finds applications in climbing robots, enhancing their compliance and capabilities to scale various surfaces.^[^ ^9^, ^18^, ^26^, ^30^ ^]^ Rock climbing, as a form of locomotion, demands precise control over attachment forces and high movement accuracy. However, VMC faces challenges in meeting both of these requirements simultaneously.

**Figure 1:**
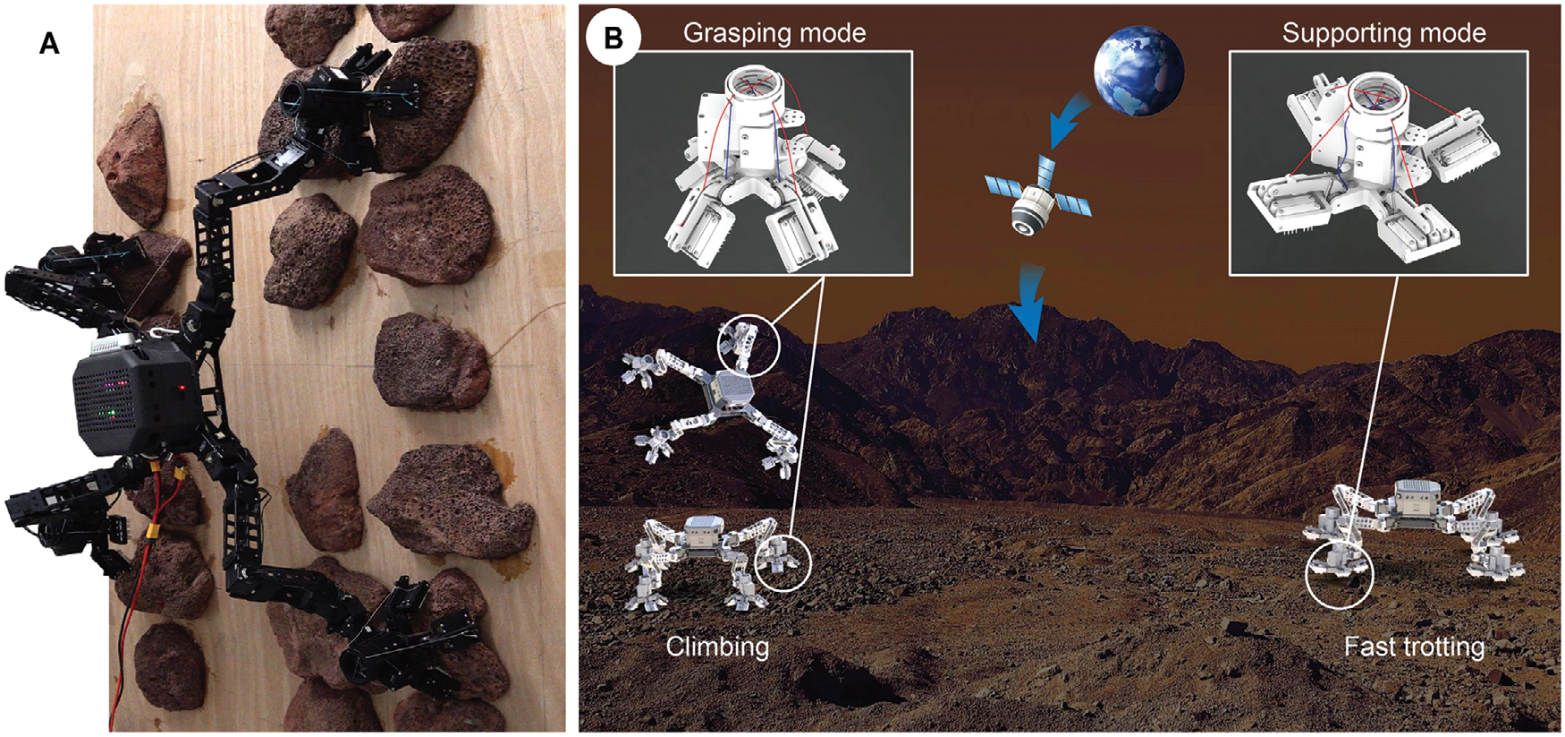
MARCBot. A) Prototype. B) Conceptual illustration of MARCBot.

In this study, we developed a novel rock‐climbing robot named MARCBot, as shown in **Figure** **1**A and Movie S1 (Supporting Information). We compared various robotic leg configurations and designed the quadrupedal mechanism of MARCBot using screw theory that can complete free‐climbing. A semi‐passively actuated, structure‐adaptive, and spiny (SPASAS) gripper with multimodal and morphing functionality, and high attachment performance was designed and applied based on mechanical intelligence and hybrid bioinspiration. In addition, we proposed a quasi‐whole‐body control (Q‐WBC) method for the optimal attachment wrench distribution based on the dynamics model. To validate the designed robot configuration and proposed control algorithm, we conducted several function and performance tests using MARCBot. In a gravity environment, the robot can climb freely on vertical surfaces using discrete rocks as footholds owing to its grippers, configuration, and controller. It can also achieve fast walking on complex terrains with small inclination angles. Figure 1B shows the conceptual illustration of MARCBot in planetary exploration by using versatile locomotion. The main contributions of this study are:

1. We proposed the concept of hybrid bioinspiration and designed a multimodal spiny gripper with fast attachment and detachment, high attachment strength, and adaptability to various terrains.
2. We proposed a configuration of a free‐climbing robot with four 5‐DOF limbs and a heterogeneous joint arrangement, thereby reducing the leg and overall mass of the robot.
3. We proposed the Q‐WBC to optimize attachment wrench distribution that can be applied to a multi‐legged climbing robot, thereby reducing the joint torque and required attachment wrench.
4. MARCBot demonstrates multiterrain adaptability in extreme environments using hybrid bioinspiration. MARCBot stands out as the sole robot capable of climbing on rock surfaces and trotting in complex terrains without switching end effectors.

## 2 Result

### 2.1 System Description of MARCBot

**Figure 2:**
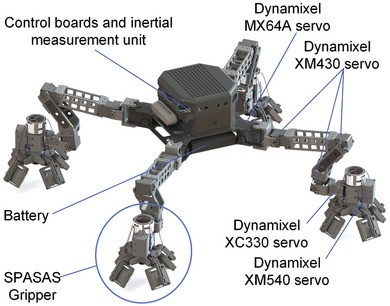
Overall design of MARCBot.

The overall design of the MARCBot is shown in **Figure** **2**. Each leg has a spiny gripper for stance and attachment. The legs were arranged symmetrically around their circumference. The body length is 0.2 m and the leg extension is 0.39 m. Each leg has five joint motors and one gripper motor. A heterogeneous design was used for different joints in the robot based on the dynamics model to reduce the inertia of the limbs. The weight of the robot is 4.8 kg.

In this section, the robot system is introduced, focusing on three key technologies involved in climbing robots: the attachment device, configuration design, and control method.

#### 2.1.1 Hybrid Bioinspired Spiny Gripper

**Figure 3:**
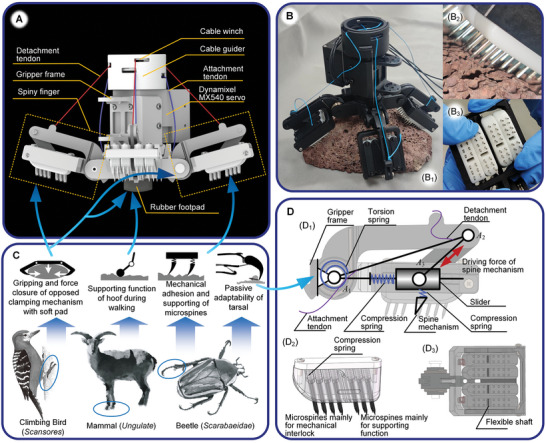
SPASAS gripper with hybrid bioinspired design concept. A) Components of the gripper. B) Prototype of the SPASAS gripper. B_1_) is the overall view. B_2_) illustrates the contact between the microspines and the rock. B_3_) illustrates load sharing between different spiny modules. C) The design was inspired by the beetle tarsal, bird claw, and the mammalian hoof. The supporting function mimics the mammal hoof. The opposed clamping mechanism was inspired by the bird claw. The spine mechanism and attachment process emulate those of beetles. D) Design of the phalange. D_1_) shows the phalange mechanism. D_2_) illustrates the linear‐constrained spine mechanism. D_3_) depicts the flexible shaft for load sharing.

The existing attachment devices used by climbing robots often have limited functionality and are restricted in their locomotion abilities on diverse terrains in mountainous and rocky environments. Moreover, the motion of many attachment devices is complex and slow. To address these limitations, we abstracted and combined the features and functions of the beetle tarsal chain, arboreal bird claw, and ungulate hoof to develop a versatile attachment device, as illustrated in **Figure** **3**A–C. The proposed gripper incorporates SPASAS features that provide strong adhesion, known as the SPASAS gripper. It can rapidly attach to and detach from the rock surface within 3 s. Additionally, it provides a multimodal function using metamorphosis that enables the robot to climb rocks and trot on the ground. These capabilities are realized through the hybrid bioinspiration of spine mechanisms, opposed clamping mechanisms, a compliant footpad, phalange mechanisms, and a specialized drivetrain using.

Many animals use claws and spines for temporary attachment while in motion, allowing them to interlock with asperities on rough surfaces. Biomimetic spine mechanisms inspired by beetles with passive adaptability are well‐suited for attachment in rough terrains.^[^ ^9b^ ^]^ The “linear constrained spine mechanism,” which consists of multiple spines with independent suspensions, is designed for attachment to rock surfaces. It offers a higher attachment probability than the other spine mechanisms.^[^ ^31^ ^]^ The SPASAS gripper utilizes linear‐constrained spine mechanisms. There are 56 1‐mm diameter spines in each phalange and a total of 224 spines in the gripper. The spacing between the microspines and the necessary sliding distance were obtained from^[^ ^32^ ^]^ whereas the other design parameters were obtained from ref. [31]. More details about the spiny module can be found in Section S1.1 (Supporting Information).

The gripper has two opposed clamping mechanisms that can transform the shear forces of the spines to normal attraction. Opposed clamping mechanisms are commonly found in the claws of arboreal birds, which are capable of gripping surfaces of different curvatures and generating normal attachment force by squeezing the attached substrates.^[^ ^21^, ^33^ ^]^ The rubber footpad with a small curvature and hollow structure at the bottom of the gripper can touch the ground when the phalanges are pulled up. This footpad, combined with a 2‐DoF ankle joint (the fourth and fifth joints of the robot leg) which is similar to the pastern and coffin joints of hoofed animals, can prevent slipping during walking. This design of morpho‐functionality referred to the studies.^[^ ^34^ ^]^ Consequently, the robot can move quickly on flat ground without replacing the end‐effector.

The attachment process of the single phalange was designed to mimic that of the beetle tarsal, as shown in Figure S1 (Supporting Information) of Supporting Information, which enables it to adaptively grip nonstructural rock surfaces without complex control. The beetle claw can search for asperities that can be attached to the shear direction of the rough substrate after the claw tip contacts the substrate, and its motion in the normal direction is constrained. Once a feasible asperity is captured, the muscle inside the tarsal contracts and drives the claw to interlock with the asperity. Because of the underactuated mechanism and elastic components of the tarsal, the beetle claw has strong adaptability to rough surfaces.^[^ ^35^ ^]^ The drivetrain of the gripper phalange, as shown in Figure 3D1, was designed to transform the functions of the beetle tarsal. The driving member is bar *A*_1_ *A*_2_, and the other bars are the driven member. When the attachment cable tendon is tightened, a driving force is applied to *A*_1_ *A*_2_ for grasping. The driving force is then transferred to the spine mechanism slider by *A*_2_ *A*_3_. Under the force from *A*_2_ *A*_3_ and two preloaded compression springs at its back, the spine mechanism did not produce tangential motion along the phalanges until it contacted the rock surface. When the spine mechanism contacts the rock, it moves tangentially under the driving force generated by *A*_2_ *A*_3_, thereby increasing the likelihood of capturing the surface asperities within the microspine tips.

A 0.6‐mm diametral steel flexible shaft passes through the four spine modules and *A*_2_ *A*_3_ as shown in Figure 3D2. When a tangential force is applied to a specific array of microspines, the flexible shaft can be deformed, resulting in a tangential relative motion between the spine modules as shown in Figure 3D3. This design allows more spines to be attached to the surface protrusions and addresses the problem of lack of tangential compliance in the previous linear constrained spine mechanisms.

**Figure 4:**
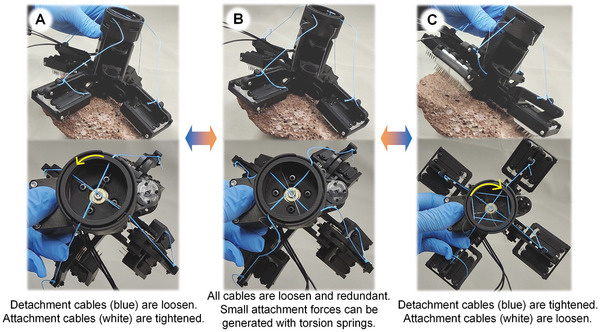
The attachment and detachment of SPASAS gripper. The blue and white tendons represent the detachment and attachment cables, respectively. A) Engagement state. When the winch is turned counterclockwise, the phalanges are passively driven by torsion springs first. Then the detached cables are pulled tightly to enhance the grasping force. B) Natural state. The four cable tendons are all loose and redundant. C) Disengagement state. When the winch is turned clockwise, each phalange is pulled up, and the gripper is detached.

The attachment and detachment processes are illustrated in Movie S2 (Supporting Information) and **Figure** **4**. The gripper is powered by a single servomotor connected to a winch that pulls the cable tendons. The winch has a hollow structure with four layers of grooved surfaces, as shown in Figure S2 (Supporting Information). Two attachment and two detachment cable tendons are linked to the opposite side of the phalange through the grooves of the winch.

Before attachment, the footpad will contact the rock surface first. At this point, the footpad serves as a cushion. During the attachment, the footpad also maintains contact. A precompression attachment strategy using soft pads, inspired by bird talon gripping and attachment, is incorporated into the attachment process. The attachment force analysis in Section S1.2 (Supporting Information) can demonstrate that the contact between the footpad and the rock surface can expand the feasible region of loads that the gripper can withstand.

In the attachment process, the torsion spring out of *A*_1_ passively drives phalange gripping first and none of the cables are tightened. The design in this case was inspired by the studies in refs. [24, 36]. The benefit of this manner is that no active control is required and each phalange can make contact and form an attachment to the rock surface. However, because the grippers in refs. [24, 36] are entirely passively driven, they may not generate sufficient attachment force and lack active adjustment capabilities. After the attachment is formed driven by passive torsion springs, the winch continuously tightens the attachment cable, enhancing the attachment force generated by the spine mechanisms, as shown in Figure 4A. The serial elastic element, which consists of the cable tendon, torsion spring, and compression spring, deforms significantly during attachment, allowing load sharing between the two clamping mechanisms.

During disengagement, the winch is turned clockwise and returned to the natural state first. The four cable tendons are all loose and a redundant 3.5 cm of each cable length, as shown in Figure 4B. The purpose of this redundancy is to achieve a greater range of motion in the phalange joints and passive adaptability through the use of torsion springs. Then, the detachment cable is tightened to drive the spiny modules to move outside the gripper, as shown in Figure 4C and Figure S3 (Supporting Information). By transferring power via the planar linkage system, detachment failures caused by the tangential force component along the inner side of the gripper when a similar attachment device applies detaching force directly to the phalange via the cable are avoided.

Consequently, the designed attachment device has the advantages of a small number of actuators, the ability to adapt to various terrains without requiring complex control, load sharing, and metamorphosis for gripping and walking.

#### 2.1.2 Mechanism and Configuration for Multimodal Function

**Figure 5:**
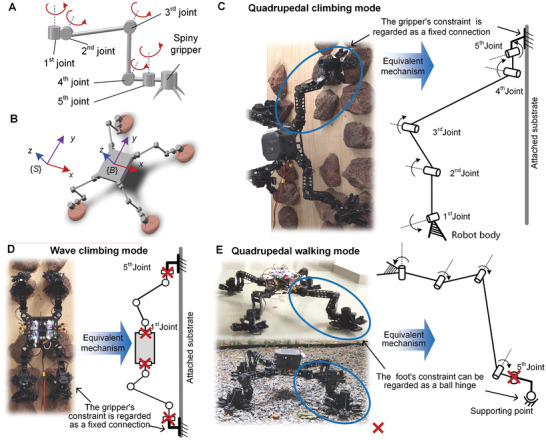
Mechanism design and multiple locomotion modes of MARCBot. A) Mechanism of single leg. B) Mechanism of robot. C) Quadrupedal climbing mode for general climbing locomotion. D) Wave climbing mode with redundant DOFs in a 2D plane. E) Quadrupedal walking mode for trotting in flat or complex terrains.

A 5‐DOF leg mechanism based on the sprawling‐type configuration of quadrupedal robots was used in MARCBot, as shown in **Figure** **5**A,B. The sprawling‐type leg configuration ensured that the robot's body was closer to the supporting surface, resulting in a smaller overturning moment. In addition, this configuration offers a larger workspace, allowing the robot to choose more footholds.

In certain climbing robots, a fourth joint parallel to the third joint is added to the sprawling‐type leg configuration.^[^ ^32^, ^37^ ^]^ This addition allows the robot to achieve planar translational motion and move forward in a singular configuration when the axes of the second and third joints of all stance legs are parallel as shown in Figure S4 (Supporting Information). When multiple grippers are attached to the surface, the robot body has three DOFs (translations along the *y*‐ and *z*‐axes and rotation about the *x*‐axis in frame {*B*} in Figure S4 (Supporting Information). The robot can only move along the *z*‐axis when it is not in this singularity configuration. However, in a general configuration, this type of leg does not enable the robot to complete the climbing motion, resulting in a limited choice of footholds.

To enable the robot to perform flexible climbing movements, it is necessary to further increase the number of DOFs in the robot's single leg. MARCBot has five DOF legs, with the fifth joint positioned before the gripper parallel to the first joint as illustrated in Figure 5B. An analysis of robot mobility based on screw theory, presented in Section S1.3 (Supporting Information), demonstrates that the robot can perform the spatial translation and rotation along the *z*‐axis in its default configuration as shown in Figure 5B. This means that the robot can move its body in any direction and change its yaw angle while climbing. Furthermore, in some singular configurations, the robot is only limited to rotation along the *y*‐axis.

An analysis of the mobility of the robot revealed that the MARCBot design can effectively meet the requirements for free climbing. This specific robot configuration has the fewest DOFs required to execute such a maneuver on an inclined terrain. Increasing the number of active DOFs in the limbs of the robot increases the flexibility of its movement. However, this increases the mass of each leg. Consequently, the dynamic performance of the robot when walking on flat ground is reduced, making all‐terrain adaptation more challenging. Moreover, the presence of a redundant DOF complicates the design process and limits the availability of a suitable workspace.

Given the diverse and complex terrains found in the field and planetary environments, such as Mars—a rocky planet with surfaces predominantly covered by volcanic rocks and home to Olympus Mons, the largest volcano in the solar system. Mars also features vast expanses of sand and gravel. Consequently, planetary exploration robots should ideally be equipped with multimodal locomotion capable of navigating effectively across various terrains. The MARCBot's locomotion modes are divided into three:

*Quadrupedal Climbing Mode*: In this mode, the robot uses all its joints for actuation when attached to and climbing steep rock faces as illustrated in Figure 5B, Movies S3 and S4 (Supporting Information). It utilizes the SPASAS grippers for secure grasping and attachment, generating a fixed connection with the rock. This mode offers versatility with its ability to take large steps and utilize numerous footholds. However, it requires significant joint torque, resulting in high energy consumption.

*Wave Climbing Mode*: Here, a single leg is driven by 3 active DOFs (the second, third, and fourth joints), while the remaining joints are locked as illustrated in Figure 5C and Movie S5 (Supporting Information). The axes of these active joints are aligned both with each other and with those in the other limbs. This alignment facilitates coordinated wave movements reminiscent of an inchworm. The robot's motion is restricted to a 2D plane, but it also possesses redundant DOFs in this plane, enhancing its flexibility. This mode can reduce joint torque and energy consumption. However, when this mode is used, the feasible footholds are limited.

*Quadrupedal Walking Mode*: This mode is employed on flat or uneven ground, where all gripper fingers are lifted, allowing direct contact through the footpads as illustrated in Figure 5D and Movie S6 (Supporting Information). The foot‐ground interaction can be equivalent to a ball hinge connection. The fifth joint is locked, and each leg has four DOFs, which facilitates high‐efficiency and high‐speed movement. When navigating soft terrains like gravel and sand, the robot may risk tumbling and slipping. If there is a tendency for the robot to overturn, fingers can make contact with the ground. Consequently, the microspines can serve as additional support to enhance stability and improve attachment by penetrating gaps in the sand or gravel, thus preventing slipping.

#### 2.1.3 Q‐WBC to Optimize Attachment Wrench

Typically, the multi‐legged system input is represented by contact forces, which can be obtained using optimal control methods and subsequently mapped to the joint space.^[^ ^38^ ^]^ Climbing robots differ from conventional legged robots owing to the special constraints between the attachment devices and contact surfaces, which not only include constraint forces but also include constraint moments. Furthermore, climbing robots often employ heavy end effectors and actuators with large reduction ratios for large driving torques. This distinguishes them from legged robots, which commonly utilize a quasi‐direct drive configuration without end effectors. Consequently, the whole‐body control methods for quadruped robots cannot be applied to climbing robots, primarily due to the unique dynamic model and the inability to directly control torques in climbing robots.

Here, the forward kinetostatics method in^[^ ^21^, ^32^ ^]^ was used to analyze the wrench space of the spiny gripper in this study. The wrench space was then reduced to obtain the constraint condition for the attachment wrenches. The optimal attachment wrench distribution is based on QP and the whole‐body dynamics model. A current‐based position controller was used to control the joint position and the corresponding maximum current, which can introduce compliance to the climbing locomotion and mediately control the attachment wrenches. This method is referred to as Q‐WBC here and is suitable for climbing robots with special end constraints and motors with a high reduction ratio.

**Figure 6:**
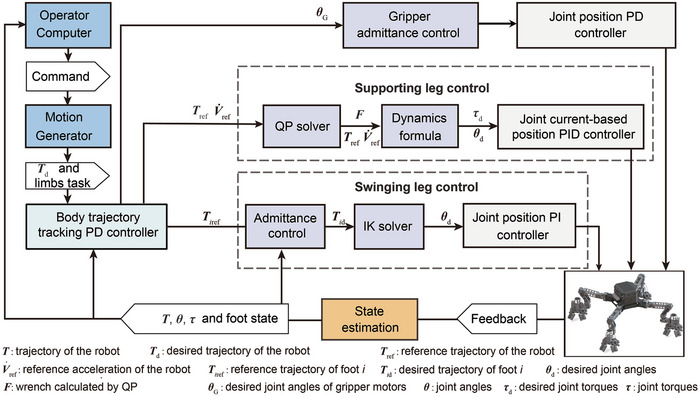
Control method of the MARCBot. The controller can be generally divided into motion planning, body trajectory control, gripper control, supporting leg control, swinging leg control, and state estimation modules.

**Figure** **6** illustrates the control architecture. The primary tasks of the motion generator are gait and trajectory planning. The proportional differential (PD) controller on *SE*(3) was used to track the desired body trajectory. The swing leg was controlled using admittance control and inverse kinematics (IK). The joint variables were solved using the IK model and proportional integral (PI) control of the servo in the joint space was employed to ensure high positional accuracy of the gripper. The stance leg control loop produces the desired optimal attachment force based on the kinematic variables of the robot and the dynamics model. The mapping relationship with the joint torque was established using dynamics to output the desired force feedforward, which was then converted to the desired current to the servos using the current coefficient in a quasi‐static climbing motion by a proportional integral differential (PID) controller. The state estimator is based on the methods in refs. [38, 39]. The extended Kalman filter was used to calculate the robot location and velocity by combining the feedback from the inertial measurement unit (IMU), the motion state of the limbs, and joint encoder information. The details about the body PD controller, kinematics and dynamics models can be found in Sections S1.4–1.6 (Supporting Information).

The optimal objective in QP is set in three parts, namely minimizing the difference between the resultant attachment wrench from the grippers and the resultant external wrench required for robot motion, minimizing the norm of the input attachment wrench, and minimizing the difference between the input attachment wrench in the current cycle and that in the previous cycle. It can then be transformed into a QP problem as follows:

(1) $$\begin{array}{cc} J & ={\left(\mathrm{AF}-B\right)}^{T}W_{1}\left(\mathrm{AF}-B\right)+F^{T}W_{2}F\\ & \;+\;{\left(F-F_{\mathrm{prev}}^{*}\right)}^{T}W_{3}\left(F-F_{\mathrm{prev}}^{*}\right) \end{array}$$

where $F\in R^{24\times 1}$ is the attachment wrench, which includes the attachment forces and torques of grippers, $A\in R^{6\times 6n}$ denotes the matrix to transform F to space frame {*S*} and $F_{\mathrm{prev}}^{*}$ represents F in the last cycle. $W_{1}$, $W_{2}$, and $W_{3}$ are positive‐definite weight matrices, which can adjust the proportion of the weights of the terms (AF−B), F, and $\left(F-F_{\mathrm{prev}}^{*}\right)$, respectively. The cost function can be expressed in the standard form of the QP problem as follows:

(2) $$\begin{array}{c} J^{\prime }=F^{T}HF+F^{T}c\\ \mathrm{Subject}\mathrm{to}\left\{\begin{array}{c} C_{E}^{T}F+c_{E}=0\\ C_{I}^{T}F+c_{I}\leq 0 \end{array}\right. \end{array}$$

where $H=2A^{T}W_{1}A+2W_{2}+2W_{3}$ and $c=-(2A^{T}W_{1}B+2W_{3}F_{\mathrm{prev}}^{*})$. The equality constraint can be obtained using the dynamics formula methods. However, the inequality constraint cannot be easily determined and can be obtained from the attachment wrench of the SPASAS gripper.

### 2.2 Experimental Results of the Robot

**Figure 7:**
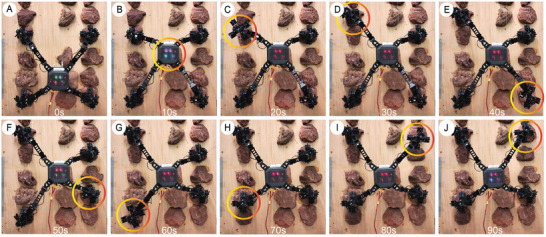
Time‐lapse photographs of climbing vertical surface. A) Initial state. B) Moving the body. C) Left front (LF) leg swinging. D) LF leg grasping. E) Right rear (RR) leg swinging. F) RR leg grasping. G) Left rear (LR) leg swinging. H) LR leg grasping. I) Right front (RF) leg swinging. J) One gait is completed after RF leg grasps the rock.

A series of climbing and walking tests were conducted to validate the proposed design and methods. MARCBot demonstrated a high level of climbing and terrain adaptability as it was able to climb on both 70° inclined and 90° vertical surfaces. To evaluate the climbing performance of MARCBot, we designed a test device in which the main frame consisted of aluminum profiles. The foothold of the robot consisted of basalt, a common rock found on Earth, Moon, Mars, and other planets. Epoxy resin was used to bond the basalt to a wooden substrate, which was fixed to the main frame of the testing platform as shown in **Figure** **7**.

When oriented perpendicular to the substrate, the diameter of the circumscribed circle of three points around the rock can reach 18.14 ± 2.44 cm, while the diameter of the smallest inscribed circle of three points is 12.18 ± 3.78 cm. The maximum height, relative to the base substrate is 7.2 ± 2.9 cm. If the rocks were restored to their uncut state, the diameter of the sphere that could envelop all the rocks would be 21.2 cm. The linear roughness *R*_a_ of these rocks is 506.5 ± 153.9 µm and their root‐mean‐square roughness *R*_q_ is 570.3 ± 174.3 µm.

Climbing on discrete foothold surfaces proved to be more difficult than climbing on continuous foothold surfaces, such as in the experimental setup of LEMUR 2 B and LEMUR 3.^[^ ^26a^ ^]^ In the flat walking test, the motion function and performance of MARCBot were tested on various support planes, including gravel surfaces, muddy ground, grass, and stone ground.

#### 2.2.1 Vertical Surface Climbing

The climbing ability of MARCBot was evaluated using the aforementioned test platform. The test surface was placed at an angle of 90°. The Q‐WBC method based on the dynamics model and QP mentioned above was used in this experiment. Because of the excessive internal forces and load of the attachment device, the robot was unable to complete the climb on the 90° surface using CLIK control and VMC methods. In the experiment, the cycle time of a single gait for climbing was 90 s, and the displacement of the moving body was 0.22 m. To improve the stability of the robot during locomotion, the body motion was decoupled from the single‐leg swing. The motion process is illustrated in Figure 7 and Movie S3 (Supporting Information). The robot first moved its body into a quadrupedal attachment state. The four legs then completed the detachment, swinging, and attachment phases, thereby completing the gait cycle. During the swinging phase, the robot leg achieved a controllable contact force and self‐adapted to the rock shape using admittance control. While climbing, the movement of the robot was mostly smooth. However, when switching from a quadrupedal attachment to a tripedal attachment, the configuration relationship between the attachment device and the surface to be attached changes because of the sudden change in the force applied to each gripper. This abrupt change in attachment force produced errors in the motion of the individual limbs of MARCBot.

**Figure 8:**
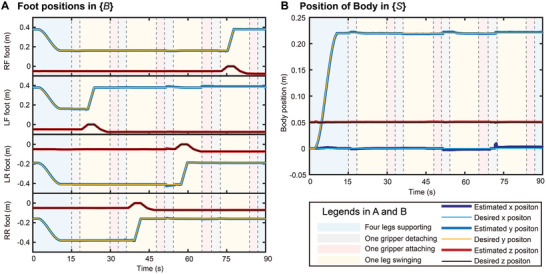
Result of vertical surface climbing. A) Foot positions in body frame {*B*}. B) Body position in space frame {*S*}.

The displacement profile of each limb and the displacement of the body after state estimation by the legged robot odometer are shown in **Figure** **8**A,B, respectively. To further highlight the state of each limb, we used colored graphical strips to represent the various stages of motion such as supporting, swinging, disengaging, and attaching. The locomotion of each limb tracked the desired trajectory using the proposed controller, as shown in Figure 8A. The climbing speed of the robot on the vertical surface was 0.15 m min^−1^, and the body length moved per minute (BL) was 0.73 min^−1^.

It can be observed from the results in Figure 8B that the actual position of the robot followed the desired position curve well, which demonstrates the feasibility and accuracy of the proposed Q‐WBC. The errors generated by the displacements in each direction during movement were primarily caused by detachment and attachment of the gripper. The abrupt changes in the forces on each limb of the robot are due to the complex morphology of the attached surface and significant uncertainty. Furthermore, the single limb of the robot, which uses the admittance control algorithm to search the terrain along the normal direction, perturbs the robot when it comes in contact with the rock surface.

#### 2.2.2 Incline Surface Climbing

**Figure 9:**
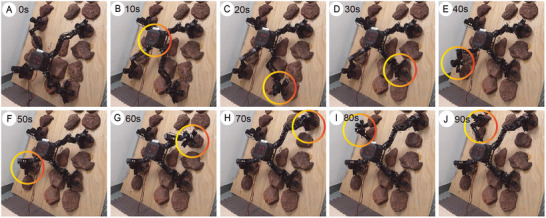
Time‐lapse photograph of climbing 70° incline. A) Initial state. B) Moving the body. C) RR leg swinging. D) RR leg grasping. E) LR leg swinging. F) LR leg grasping. G) RF leg swinging. H) RF leg grasping. I) LF leg swinging. J) One gait is completed after LF leg grasps the rock.

The climbing test platform was set at an inclination of 70°, and the robot was subjected to climbing motion tests on a large inclined surface. The CLIK control, VMC, and the Q‐WBC proposed in this study were used in these tests, and their respective results were compared. The details about CLIK control and VMC methods can be found in Section S1.7 (Supporting Information). In tests, the robot used the same gait parameters to complete the climbing motion: the fuselage moved 220 mm in a single gait cycle, the gait cycle lasted 90 s, the climbing speed of the robot was 0.15 m min^−1^, and BL was 0.73 min^−1^. **Figure** **9** and Movie S4 (Supporting Information) illustrate the climbing process.

In five tests, the robot successfully executed a gait cycle on a 70° slope incline using the proposed Q‐WBC, achieving a 100% success rate. In contrast, using CLIK control, the robot completed the gait cycle in three out of five climbs. Similarly, with VMC control, the robot completed the gait cycle only three times. Therefore, compared to the other two methods, the Q‐WBC proposed in this study is more reliable.

**Figure 10:**
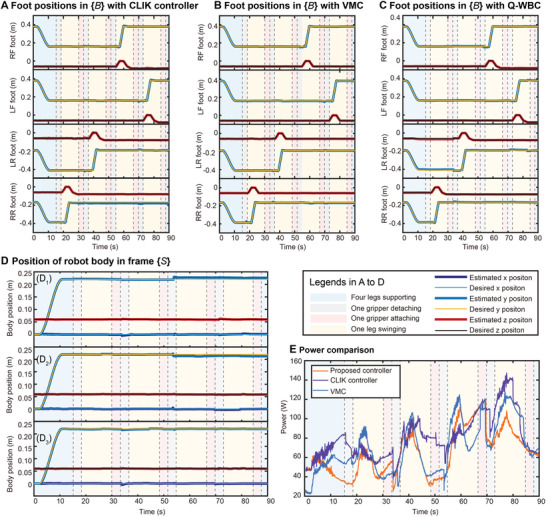
Result of 70° surface climbing. A) Foot positions in body frame {*B*} when using CLIK controller. B) Foot positions in body frame {*B*} when using the VMC. C) Foot positions in body frame {*B*} when using the proposed Q‐WBC. D) Comparison of body positions in space frame {*S*}. D_1_) illustrates the body position when using the CLIK controller, D_2_) illustrates the body position when using VMC, and D_3_) illustrates the body position when using the proposed Q‐WBC. E) Comparison of power consumption.

**Figure** **10**A–C shows the displacement curves of each foot in body frame {*B*} and the body position during the climbing process is shown in Figure 10D. When the Q‐WBC was used, the body trajectory was smoother and the error during motion was smaller than those of the CLIK control and the VMC. Because of the overconstrained state of the MARCBot mechanism, which resulted in excessive internal forces, it was difficult to adjust the actual position of the robot's body to the desired position using the CLIK control, leading to a steady‐state error. VMC method is simple and efficient, but it does not offer high control precision. Consequently, during motion, VMC does not effectively follow the trajectories. This motion error can prevent the robot feet from reaching the specified attachment points, leading to attachment failures. In contrast, the proposed Q‐WBC achieved the desired position. The maximum errors obtained using the CLIK control, the VMC, and the Q‐WBC were 11.12, 8.54, and 6.64 mm, respectively, representing reductions of 40.29% and 22.25% by using the Q‐WBC.

Figure 10D illustrates the total power consumption of all joints during climbing using three different methods: the proposed Q‐WBC method, CLIK control, and VMC. The figure depicts three lines representing the power consumption for each method. The colored strips have the same meaning as described above. Figure 10D demonstrates that in most cases, the overall power of the robot joints was lower when the proposed Q‐WBC was used for climbing. This indicates a reduction in the power of the robot joints as well as the internal forces between the limbs. It also suggests that the load on each gripper was smaller. During testing with the CLIK control method, the average power consumption of the mechanical system was 80.84 W, with a maximum of 147.65 W. With VMC, the values were 69.71 and 128.03 W, respectively. In contrast, using the proposed Q‐WBC method, the values were 64.65 W for average power and 120.29 W for maximum power, yielding cost‐of‐transport reductions of 20.03% and 6.05% compared to the other methods. These results demonstrate that the proposed Q‐WBC can improve the tracking performance of the robot motion, reduce the peak and average power, and decrease the overall loads on the joints and internal forces, resulting in a more reliable attachment of each gripper compared with using the CLIK control and the VMC.

Figure 10D also shows that when the gripper was detached, the internal force of the robot was released and the joint loads decreased. In contrast, when the gripper was attached, the overconstraint of the robot worsened, which increased the internal force and overall power of the joints. However, using the Q‐WBC can mitigate this trend to a certain extent. The above results also support the hypothesis that the overconstraint of the robot during attachment increases its overall internal force and joint moment. As the robot moves, deformations and slight slips occur, causing changes in the relative configuration between the gripper and the rocks. However, the proposed control algorithm assumes a solid connection between the attachment device and the rock, which may result in a gradual enhancement in the overall overconstraint of the robot.

#### 2.2.3 Walking on the Ground

**Figure 11:**
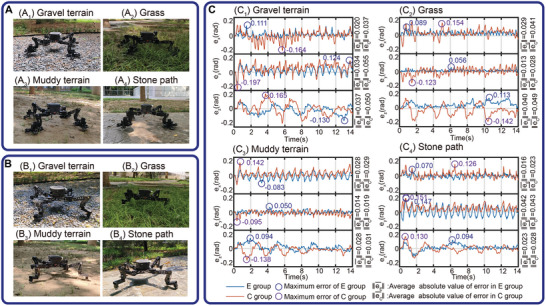
Walking tests of MARCBot. A) Trotting with the SPASAS grippers in various terrains, that is, E groups. B) Trotting with rubber footpads in the same terrains, that is, C groups. C) Comparison of the attitude errors on the exponential coordinates of E groups and C groups.

MARCBot is highly adaptable to various terrains and can traverse complex ground as shown in **Figure** **11**A and Movie S6 (Supporting Information). This unique capability distinguishes it from other similar climbing robots. To evaluate the effect of the gripper on robot locomotion on different complex terrains, we conducted trotting tests using MARCBot. In addition, we replaced the gripper with a regular rubber foot and conducted walking tests. The tests with SPASAS grippers were designated as the experimental group (E groups), whereas the tests with rubber feet were the control group (C groups). The test data were used to evaluate the effects of the spiny gripper on the motion of the legged robot. Figure S5 (Supporting Information) illustrates the robot configurations in the E groups and C groups. After replacing the spiny gripper with a regular foot, the mass of each leg decreased by 0.41 kg. Park et al.^[^ ^40^ ^]^ utilized spine arrays on a legged robot to enhance the friction of the robot foot to prevent slipping in complex terrain. However, no research has been conducted on combining a spiny gripper with the walking ability of legged robots.

The robots were tested on gravel, grass, muddy terrain, and stone paths, as shown in **Figure** **11**A,B. A trotting gait was executed in the tests in which the diagonal legs alternated, landing on the ground for support. Each gait cycle lasted 0.7 s. The leg duty cycle was set to 0.6 such that in some instances, all the legs were in stance simultaneously. With a walking step length of 150 mm and step height of 80 mm, the desired average motion rate of the robot was 0.21 m s^–1^. The control used was based on CLIK, and the IMU measured the fuselage attitude.

Figure 11C illustrates the attitude errors in the exponential coordinates of the robot while walking on gravel, grass, muddy terrain, and stone paths using the initial pose as a baseline. The average and maximum errors for the E groups and C groups are also shown in Figure 11C. The comparison shows that the E groups exhibited lower average and maximum errors in all test scenarios, indicating that the SPASAS gripper contributed to a smoother motion of the legged robot. Consequently, it can be concluded that the robot achieved greater stability when traversing complex terrain. When the robot body tilts, the gripper fingers can touch the ground to provide additional support. Spiny grippers offer the robot a larger actual support area, which can enhance the robot's stability during walking.

The average power of the robot mechanical system measured over 20 gait cycles across the four terrains was 66.42, 69.22, 65.77, and 66.10 W for the E groups, and 55.166, 55.389, 49.929, and 50.615 W for the C groups, respectively. The E groups consumed more energy than the C groups owing to the increased leg mass and inertia. Therefore, this type of end‐effector is suitable for highly stable movement in complex environments, but not for flat terrain where efficiency is crucial.

## 3 Conclusion

We designed a quadruped rock‐climbing robot known as MARCBot for planetary exploration, field operation, and scientific investigation. The SPASAS gripper offers advantages such as a short attachment period, large attachment force, and strong surface adaptability. MARCBot uses the SPASAS gripper to achieve rapid movement on flat ground and stable climbing on vertical and inclined rock surfaces by switching modes without changing the end‐of‐foot device. This improved the terrain adaptability, climbing, and walking performances of the robot. Meanwhile, we used the spine mechanism for the first time in quadruped robot trotting in complex terrains. The spiny gripper with a rubber palm can increase the actual supporting area of the robot while maintaining its motion and avoiding slipping. Consequently, the movement of the quadruped robot on the surface of complex terrain was more stable.

The Q‐WBC method is proposed for optimal attachment wrench distribution based on the MARCBot dynamics model. This method reduces the internal force of the climbing robot while improving its compliance with the external environment. It also avoids disengagement when the payload is concentrated on a single gripper, ensuring more stable and safer motion and attachment of the robot. Furthermore, this method reduces the overall energy consumption of the robot and the peak power of the joints during climbing compared to using only the CLIK control algorithm.

A comparison between MARCBot and other climbing robots is presented in **Table** **1**. Compared to other robots, MARCBot has a better terrain adaptation ability and can move in various complex terrains without replacing the end effector. MARCBot also exhibited advanced capability in terms of climbing gravity and movement speed. More discoveries observed in the experiments are further discussed in Supporting Information. Overall, this study contributes to the advancement of climbing robots in field operations and asteroid and planetary exploration.

**Table 1:** Multi‐legged climbing robots for moving on rough surfaces.

Climbing Robot	Climbing surface	Mass (kg)	DOF per leg	Maximum speed (m min^–1^)	BL (min^–1^)	Climbing gravity	End effector	Locomotion capability
MARCBot	Up to 90° discrete natural rocks	4.8	5	0.22 (70°), 0.15 (90°), 12.84 (ground)	1.1 (70°), 0.73 (90°), 64.2 (ground)	*g*	Spiny gripper	Climbing on rocks. Trotting on gravel, sod, muddy, and stone terrains.
LEMUR 3^[^ ^2^, ^23^ ^]^	Up to 90°continuous natural rocks	35	7	0.0027	0.0067	Up to 0.38*g*	Spiny gripper	Climbing
HubRobo^[^ ^25^ ^]^	45° polymer‐made artificial bouldering holds	3	3 active + 3 passive	0.17 (45°)	0.57	Up to 0.63*g*	Spiny gripper	Climbing
SCALER^[^ ^26b^ ^]^	90° discrete polymer‐made artificial bouldering holds; 125° and 180° sectional bars	9.6	6	0.35 (90°)	1.0 (90°)	*g* (without its control board during climbing)	Spiny gripper	Climbing on forementioned surfaces; Trotting on the ground after replacing end effectors with rubber feet

Failures to climb during tests generally occur when the engagement of one limb is poor and another limb is swinging. Even when the other two limbs are well attached, they struggle to modify the attachment wrench rapidly enough at this point. As a result, limb state switching can lead to climbing failure. Improved safety in limb‐leg state switching is a major issue that needs to be addressed. In the QP‐based control, the weight of each attachment wrench of the limb about to enter the swing phase is pre‐reduced, while the weight of the other attachment wrenches is increased. This reduces the magnitude of the change in attachment force experienced by each leg during state switching. In the future, it will also be necessary to synergize the SPASAS gripper control so that when gripper slippage is detected, appropriate response and grasping techniques are utilized to prevent falling. Most of the tests are conducted in a laboratory due to cost limitations and existing experimental conditions. Although the robot is equipped with an Intel RGB‐D camera, the climbing environment used was constructed in a laboratory and all the foothold information was manually entered. Moving forward, our aim is to enhance the robustness and autonomy of MARCBot by using Simultaneous Localization and Mapping to make it applied in real‐world scenarios.

## 4 Experimental Section

### Hardware Architecture

The body of MARCBot is made of a carbon fiber plate. High‐performance nylon and aluminum alloys were used for 3D printing of the linkage of the hip, thigh, calf, and ankle for the integrated complicated structures. The hardware structure is shown in Figure 2 and Figure S6 (Supporting Information). A remote operating software was installed on a laptop and communicated with the robot via a wireless network. The main control board of MARCBot is an NVIDIA Jetson NX embedded system that runs Ubuntu 20.04. The IMU sensor is Fidei N100. Three 12.6 V 2200 mAh lithium batteries were used to power the robot. The robot actuators are Dynamixel servos, which include a direct‐current motor, reducer, controller, and sensors. Four TTL bus serial ports connected the servo controllers.

### Simplified Dynamics

The configuration of MARCBot makes its complete dynamic equation extremely complex, and the corresponding real‐time computations are weak. Additionally, accounting for nonlinear components such as viscous friction in the high‐ratio reducer motor is difficult. Unlike in typical quadruped robots, the limb masses constitute a sizeable portion of the overall robot mass (more than 70% of the total robot mass). Because of the slow motion of the rock‐climbing robot during climbing, its dynamics equation was simplified by establishing the centroid dynamics using the Newton–Euler formulation of *SE*(3).^[^ ^41^ ^]^ Its model is illustrated in Figure S7 (Supporting Information). The dynamic equation of the robot body can be written as

(3) $$\frac{d}{dt}\left(M_{B}V_{B}\right)=G_{B}+\sum F_{i,1}$$

where $M_{B}\in R^{6\times 6}$ denotes the spatial inertia matrix of the robot body in {*S*}, $V_{B}\in R^{6}$ is the generalized velocity of the robot, $G_{B}\in R^{6}$ denotes the gravitational wrench on the body, and $F_{i,1}\in R^{6}$ is the wrench of the *i* ^th^ leg's first joint. In each leg, let $F_{i,\mathrm{con}}^{i,\mathrm{end}}={\left(\begin{array}{cccc} \tau_{ix} & \tau_{iy} & \tau_{iz} & \begin{array}{ccc} f_{ix} & f_{iy} & f_{iz} \end{array} \end{array}\right)}^{T}$ $\in R^{6}$ denote the contact wrench acting on the gripper in the *i* ^th^ leg. All the attachment wrenches from legs can be rewritten as

(4) $$F=\left(\begin{array}{cccc} {\left(F_{1,\mathrm{con}}^{1,\mathrm{end}}\right)}^{T} & {\left(F_{2,\mathrm{con}}^{2,\mathrm{end}}\right)}^{T} & {\left(F_{3,\mathrm{con}}^{3,\mathrm{end}}\right)}^{T} & {\left(F_{4,\mathrm{con}}^{4,\mathrm{end}}\right)}^{T} \end{array}\right)$$

Because of the slow speed of the robot while climbing, only the gravity of each leg was considered in quasi‐static conditions. The Newton–Euler method can be used to express the dynamics equation of the body as follows:

(5) AF−B=0

where A is related to the positions and attitudes of the grippers, which can convert the wrenches exerted on each leg to the wrench exerted on the robot's body, and B is related to the acceleration of the robot's body, configuration of each leg, and gravity. For a detailed derivation, please refer to Section S1.5 in Supporting Information.

### Wrench Constraints in QP

**Figure 12:**
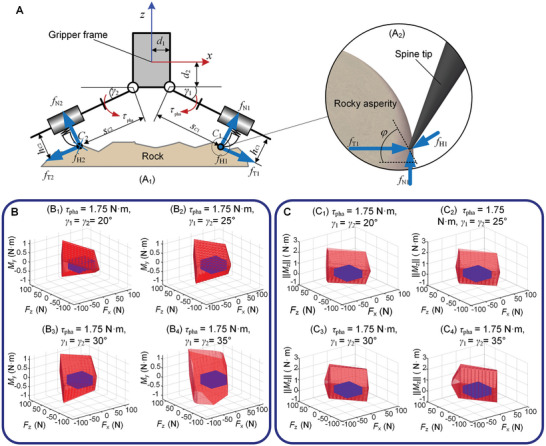
Analysis of attachment wrench constraints. A) The simplified kinetostatics model of an opposed clamping mechanism. A_1_) is the overall statics model. A_2_) is a modified local statics model between the microspine and the asperity. B) The attachment wrench space on *SE*(2) of the opposed clamping mechanism with different γ*_i_* and the enveloped constraint space. C) The components of attachment force in the x and z direction and the component of attachment torque in the z direction. In B and C, the red polyhedrons are the boundaries of the wrench spaces, and the blue cubes are the wrench constraints in the QP problem.

The forward grasp statics of such attachment devices were analyzed first, and then the grasp wrench spaces were calculated using simulation. The inequality constraint in the QP problem was then obtained. The opposed clamping mechanism is driven by the same set of cables, and it is assumed that four phalanges can achieve load‐sharing with the same driving force. **Figure** **12**A shows the simplified kinetostatics model of an opposed clamping mechanism of the gripper, in which the forces from elastic components are ignored. The planar resultant wrench $F_{\mathrm{OCMxz}}={\left(\begin{array}{ccc} f_{\mathrm{OCMx}} & f_{\mathrm{OCMz}} & \tau_{\mathrm{OCMy}} \end{array}\right)}^{T}$ generated by the opposed clamping mechanism can be written as follows:

(6) $$\left\{\begin{array}{c} f_{\mathrm{OCMx}}=f_{T1}\cos\gamma_{1}-f_{T2}\cos\gamma_{2}+f_{N1}\sin\gamma_{1}-f_{N2}\sin\gamma_{2}\\ f_{\mathrm{OCMz}}=f_{N1}\cos\gamma_{1}+f_{N2}\cos\gamma_{2}-f_{T1}\sin\gamma_{1}-f_{T2}\sin\gamma_{2}\\ \tau_{\mathrm{OCMy}}=\left(f_{N1}\cos\gamma_{1}-f_{T1}\sin\gamma_{1}\right)d_{1}+\left(f_{T1}\cos\gamma_{1}+f_{N1}\sin\gamma_{1}\right)d_{2}\\ +\left(f_{T2}\sin\gamma_{2}-f_{N2}\cos\gamma_{2}\right)d_{1}-\left(f_{T2}\cos\gamma_{2}+f_{N1}\sin\gamma_{2}\right)d_{2} \end{array}\right.$$

where $f_{T1}$ and $f_{T2}$ denote the resultant tangential forces on spines respectively in two phalanges, while $f_{N1}$ and $f_{N2}$ denote the resultant normal forces. Furthermore, $\gamma_{1}$ and $\gamma_{2}$ is the knuckle variables of two phalanges, $d_{1}$ and $d_{2}$ are the position parameters of knuckles as shown in Figure 12A_1_. The relationship between $f_{T1}$ and $f_{N2}$ can be expressed as follows:

(7) $$f_{N1}=\frac{\tau_{\mathrm{pha}}-f_{T1}h_{C_{1}}}{s_{C_{1}}}$$

where $\tau_{\text{pha}}$ is the driving torque of the phalange, and $h_{C_{1}}$, $s_{C_{1}}$ represents the distances of equivalent pressure center point $C_{1}$ of the spine array in relation to the knuckle as shown in Figure 12A_1_. Depending on the exerted force, $C_{1}$ is floating inside the range covered by the spine array. For $f_{N2}$ and $f_{T2}$, a similar relationship can be obtained.

To obtain $\tau_{z}$, the 2D spine attachment model was extended to a 3D model, as shown in Figure 12A_2_. $\tau_{z}$ is generated by the lateral forces of the spines. Take the first phalange as an example, its lateral force $f_{L1}$ is derived from the frictional component in the limit condition. The friction can be written as follows:

(8) $$f_{\mathrm{fri}}={\left({\left(f_{T1}\sin\varphi +f_{N1}\cos\varphi \right)}^{2}+{f_{L1}}^{2}\right)}^{\frac{1}{2}}$$

where φ is the local slope and the asperity at the contact point. Because φ is generally close to π/2, it can be simplified as

(9) $$f_{\mathrm{fri}}\approx {\left({f_{N1}}^{2}+{f_{L1}}^{2}\right)}^{\frac{1}{2}}$$

Thus, $f_{L1}$ can be get as

(10) $$f_{L1}=\pm {\left({f_{\mathrm{fri}}}^{2}-{f_{N1}}^{2}\right)}^{\frac{1}{2}}$$

The torque that the single opposed clamping mechanism can generate can be obtained as

(11) $$\tau_{\mathrm{OCM}z}=f_{L1}\left(s_{C1}\cos\gamma_{1}+d_{1}\right)+f_{L2}\left(s_{C2}\cos\gamma_{2}+d_{2}\right)$$

That *f*_fri_ ≤ μ*f*_T_ is used as the constraint condition, where μ is the friction coefficient.

To obtain the possible attachment wrench space of each opposed clamping mechanism, the search algorithm was used. First, different variables and numerous groups of attachment wrench were attempted in Equations (6) and (11) to obtain possible wrench spaces. After comparing these wrench space, $\tau_{\text{pha}}$ = 1.75 N m was selected as the most suitable driving torque for the gripper motor. In the case of given $\tau_{\text{pha}}$ and $\gamma_{i}$, the corresponding wrench space was searched. The boundary of the wrench space was then fitted. A cuboid was used here to represent the wrench constraint of the gripper, as shown in Figure 12B,C. Consequently, the feasible wrench space was reduced, but the solution efficiency of QP was improved by converting the nonlinear constraint to a linear constraint. Additionally, making the attachment wrench away from its boundary can also enhance the attachment reliability. The fitted convex polyhedron was tested to determine if it could realize the envelope for the space formed by the proposed force constraint inequalities. Finally, the appropriate inequality constraints were selected. Superposing the wrench spaces of the two orthometric opposed clamping mechanisms yielded the wrench space of the attachment device in Euclidean space. It was obtained that $f_{\mathrm{ix}}$ ∈ [−35 N, 35 N], $f_{\mathrm{iy}}$ ∈ [−35 N, 35 N], $f_{\mathrm{iz}}$ ∈ [−62 N, 62 N], $\tau_{\mathrm{ix}}$ ∈ [−0.25 N m, 0.25 N m], $\tau_{\mathrm{iy}}$ ∈ [−0.25 N m, 0.25 N m], $\tau_{\mathrm{iz}}$ ∈ [−1.4 N m, 1.4 N m]. So far, inequality constraint solutions in QP problem have been provided. By testing the load capacity, the attachment capacity of the gripper can cover this constraint wrench space.

### Gripper Control

A stiffness control method is used to actuate the gripper. Given that the elastic components of the SPASAS gripper (e.g., cables, springs) are primarily arranged in series, stiffness control is particularly suitable for gripper operations. The motor current feedback was utilized to calculate the corresponding drive torque by applying the current factor. When the torque reached the targeted threshold, the servo joint variable remained constant. However, if the servo driving torque surpassed the set threshold, the joint variable was reduced to release a small portion of the attachment cable. This prevented overloading and excessive energy consumption. Conversely, if the servo driving torque was insufficient, the servo kept rotating, tightly pulling the attachment cable to enhance the attachment force. To get more details about the gripper control, please refer to Section S1.8 (Supporting Information).

### Motion Planning

The algorithm for blind climbing terrain‐adaptive gait in the study^[^ ^42^ ^]^ is used to plan the robot's locomotion. The body and swing leg trajectories were planned after determining the foothold for the next gait. To avoid interference with the gripper and rock, the gripper was first lifted normally to the attached surface. It then moves tangentially along the substrate attached to the top of the next foothold. It then descends in the normal direction. When the fourth joint torque exceeds the preset threshold, the rubber pad is considered to have contacted the rock, and the grasp is activated. The robot moves only one leg at a time to ensure climbing safety, and at least three legs are attached to the rocks. The body then moves to the center of the four landing points. The trajectory was determined using cubic polynomial curve interpolation.

### Statistical Analysis

The climbing tests were conducted three times (*n* = 3). The position was estimated by an odometer based on an extended Kalman filter using the joint angles and IMU data. The power data was calculated by the current data and voltage data. The internal data of MARCBot was recorded by rosbag, which is a tool of Robot Operating System. The statistical evaluation of the data was carried out using Excel 2021 and Matlab 2020a. The results are presented as mean values.

## Conflict of Interest

The authors declare no conflict of interest.

## Supporting information

Supporting Information

Supplemental Movie 1

Supplemental Movie 2

Supplemental Movie 3

Supplemental Movie 4

Supplemental Movie 5

Supplemental Movie 6

## Data Availability

The data that support the findings of this study are available from the corresponding author upon reasonable request.
