# Supplementary material for: Intelligent Rock‐Climbing Robot Capable of Multimodal Locomotion and Hybrid Bioinspired Attachment
Source: Adv Sci (Weinh). 2024 Jul 15;11(39):2309058. doi: 10.1002/advs.202309058 (PMC11497113; doi:10.1002/advs.202309058)
Supplement: Supplementary file 1 — Supporting Information [file ADVS-11-2309058-s001.pdf]

ADVANCED SCIENCE Open Access

Supporting Information

for *Adv. Sci.*, DOI 10.1002/advs.202309058

Intelligent Rock-Climbing Robot Capable of Multimodal Locomotion and Hybrid Bioinspired Attachment

*Peijin Zi, Kun Xu, Jiawei Chen, Chang Wang, Tao Zhang, Yang Luo, Yaobin Tian, Li Wen and Xilun Ding**

Supporting Information

for

Intelligent Rock-climbing Robot Capable of Multimodal Locomotion and Hybrid Bioinspired Attachment

Peijin Zi[1†], Kun Xu[1†], Jiawei Chen[1], Chang Wang[1], Tao Zhang[1,2], Yang Luo[1], Yaobin Tian[1], Li Wen[1], Xilun Ding[1]*

1 School of Mechanical Engineering and Automation, Beihang University, Beijing, China
2 School of Electro-mechanical Engineering, Guangdong University of Technology, Guangzhou, China
*Corresponding author. Email: xlding@buaa.edu.cn
†These authors contributed equally to this work

This PDF file includes:

Figure S1 to Figure S18

S1. Supplementary Methods

S1.1. Mircospine Module Inspired by Synergistic Attachment of Beetle Tibial Spine-Tarsal Chain

S1.2. Attachment Strategy with Pre-compressed Soft Footpad

S1.3. Mobility Analysis of the Robot

S1.4. Kinematics Model of the Robot

S1.5. Simplified Dynamics Model of the Robot

S1.6. Body Tracking PD controller

S1.7. Methods of Control Groups in Climbing Tests

S1.8. Gripper Stiffness Control

S1.9. Foundation of Mathematics

S2. Supplementary Gripper Load Capacity Test

S3. Supplementary Discussion

S2.1. Feasibility of Spine Mechanism in Extreme terrain

S2.2. Visual Sensing Scheme Limitations

S2.3. Gripper Attachment Rigidity During Climbing

S2.4. Hexapod Robot versus Quadruped Robot

Supplementary References

Other Supplementary Material for this manuscript includes the following:

Movies S1 to S6

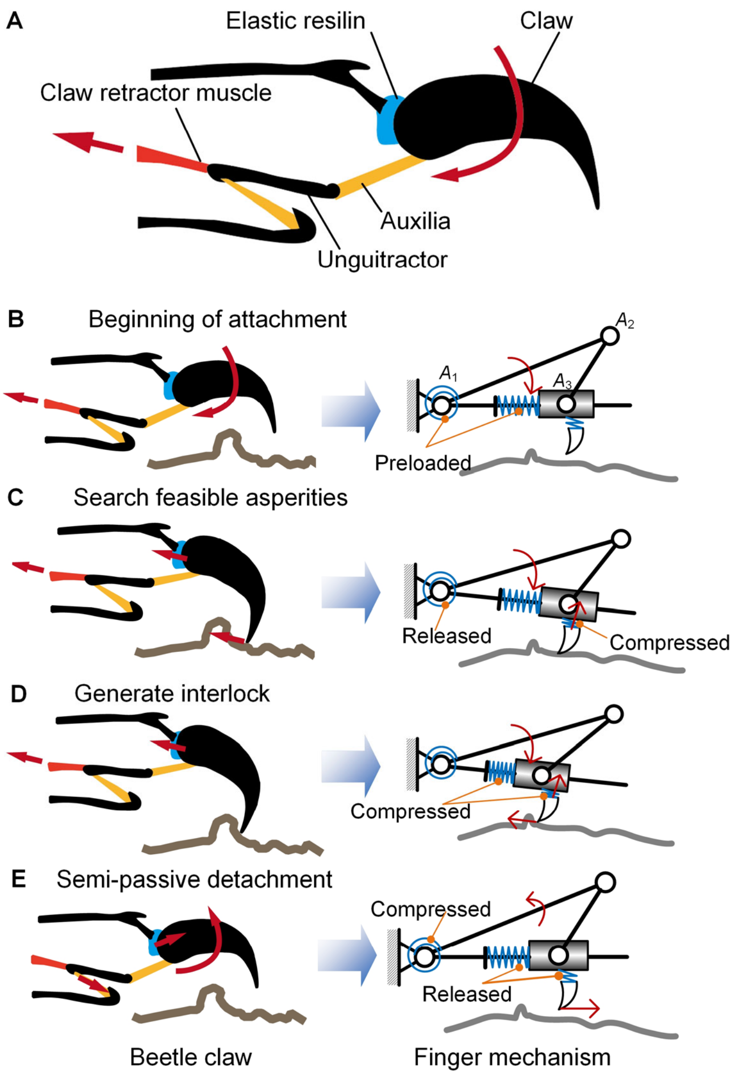

Figure S1. Bioinspired attachment process of the gripper phalange. A) Structural diagram of the beetle claw. The red arrow indicates the direction of movement. **B)–E)** illustrate the map of attachment and detachment processes from the beetle claw to the gripper finger. **B)** Before attachment, A_1A_2 is actuated and the spines close to the attached surface. **C)** The spines contact the surface, and the springs upon spines are compressed to make spines adapt to the surface shape. **D)** The spines retract to search for feasible asperities and generate attachment. **E)** During detachment, A_1A_2 is pulled off and the spine mechanism returns to initial position driven by springs and A_2A_3.

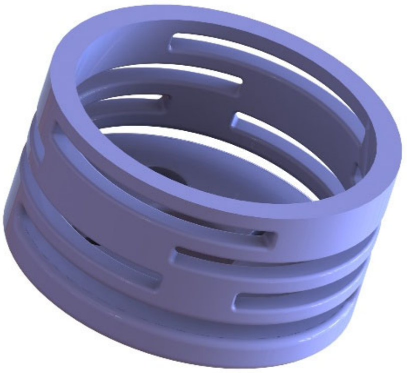

Figure S2. The specialized winch of the gripper.

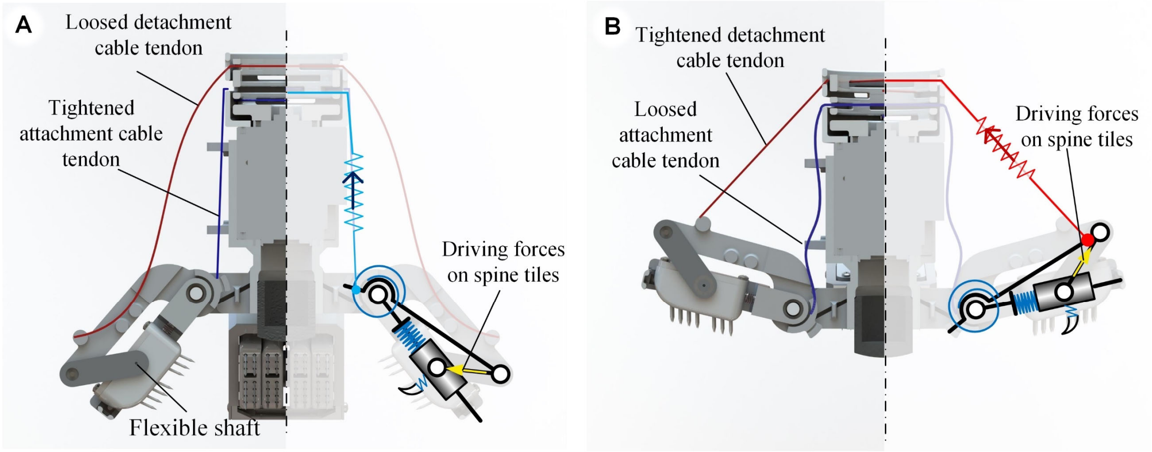

Figure S3. The drivetrain of the gripper. A) Gripper grasping. **B)** Gripper disengaging. These two figures are cross-sectional diagrams of the gripper. The left half of each figure is a three-dimensional model of the gripper, while the right half is a schematic diagram of the gripper mechanism.

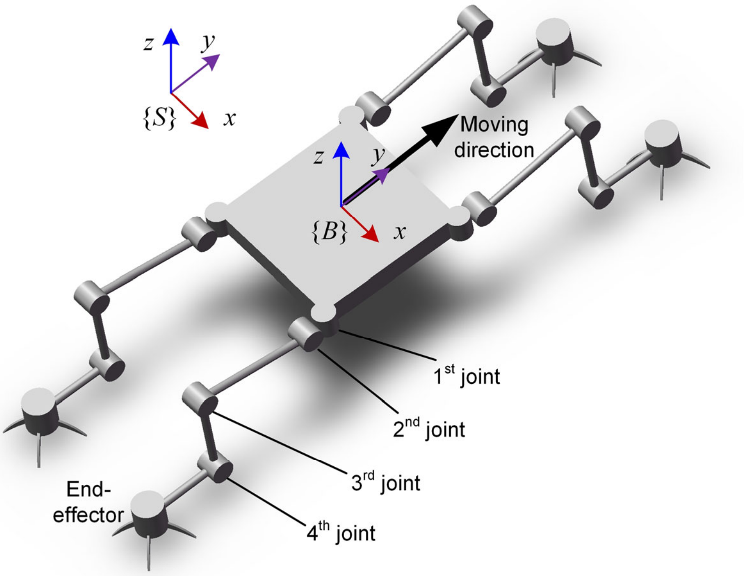

Figure S4. Mechanism of climbing robot with four-DOF legs. Robots with this mechanism can only moving forward or backward and rotate along with *x*-axis in the singlar configuration as illustration.

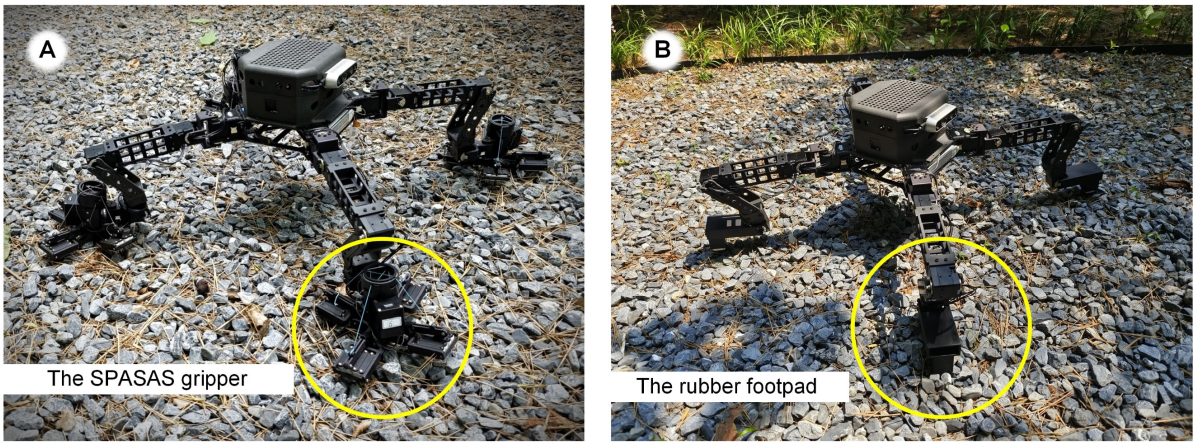

Figure S5. Prototypes in trotting tests on complex terrains. A) prototype in experimental groups. **B)** prototype in control groups.

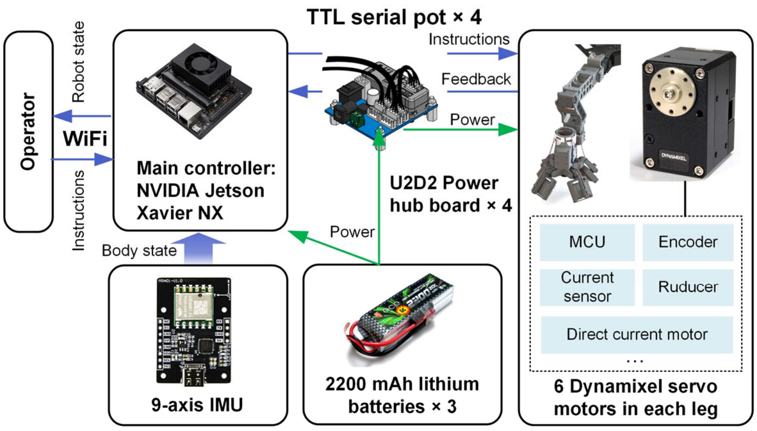

Figure S6. Hardware architecture of MARCBot.

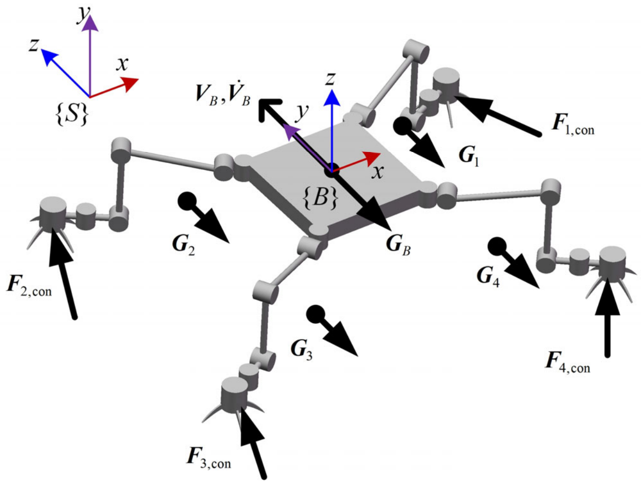

Figure S7. Simplified dynamic model.

S1. Supplementary Methods

S1.1. Mircospine Module Inspired by Synergistic Attachment of Beetle Tibial Spine-Tarsal Chain

As shown in Figure S8A, a solitary microspine lacks stability under external forces alone, tending to rotate around its tip. To prevent overturning when employing microspines for attachment, it is essential to utilize multiple contact points. Drawing inspiration from beetle locomotion, the microspine layout is modified. Typically, beetles engage the ground using the last three segments of their middle and hind limbs—the tibia, tarsus, and pretarsus (claws)—during attachment. The spines located at the ends of the tibia and tarsus of these limbs make contact with the ground. As illustrated in Figure S8B and C, the tibial spines can interlock with rough surfaces, whereas the segmented tarsal chain conforms to surface irregularities, providing both normal support and shear friction. This method of attachment presents several benefits:

(1) The normal support counters the overturning torque exerted on the limb, thus restricting potential rotational movements;

(2) If the tibial spines fail to attach sufficiently and begin to slide, the tarsal spines can compensate, preventing detachment.

Consequently, the synergistic function of the beetle's tibial and tarsal spines has inspired the design of bioinspired microspine module, as demonstrated in Figure S8D.

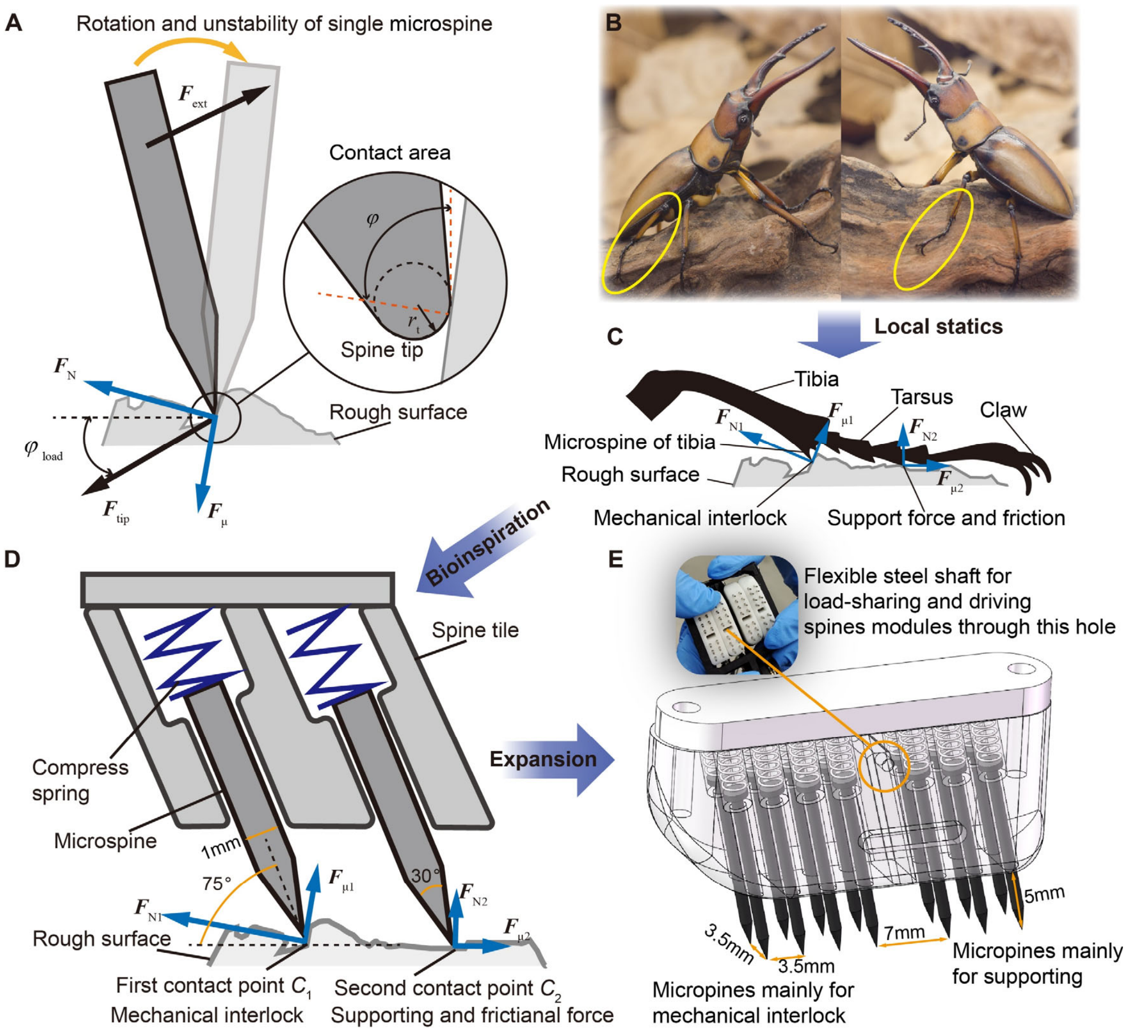

Figure S8. Bioinspired mircospine module design. A) Rotation and unstability of single microspine due to overturning moment. **B)** and **C)** Synergistic attachment of beetle tibial spine-tarsal chain. **D)** Bioinspired microspines to generate mechanical interlock and supporting, mimicking synergistic attachment of tibial spine-tarsal chain. **E)** Microspine module with expanded design.

Drawing on the cooperative attachment mechanisms of beetle tibial and tarsal spines, we have refined our spine module design, as depicted in Figure S8E. The module is partitioned into two distinct regions: the anterior region and the posterior region. The anterior region is designed to mimic the mechanical interlock function of beetle tibial spines, whereas the posterior region is intended to emulate the support force and friction characteristics of the beetle tarsus. The driving force is strategically applied between these two regions, optimizing the module's capability to counteract external forces and overturning moments. This configuration enhances the stability and functional adaptability of the microspine module.

S1.2. Attachment Strategy with Pre-compressed Soft Pad

A pre-compression attachment strategy using the soft footpad, inspired by bird talon gripping and attachment, was incorporated before attachment. A mechanical analysis of this attachment strategy is carried out. Analyzing the attachment force generated by the opposed clamping mechanism under two conditions—when the rubber footpad beneath the gripper can contact the attached surface and when it cannot—the results, as shown in Figure S9 and Figure S10, indicate:

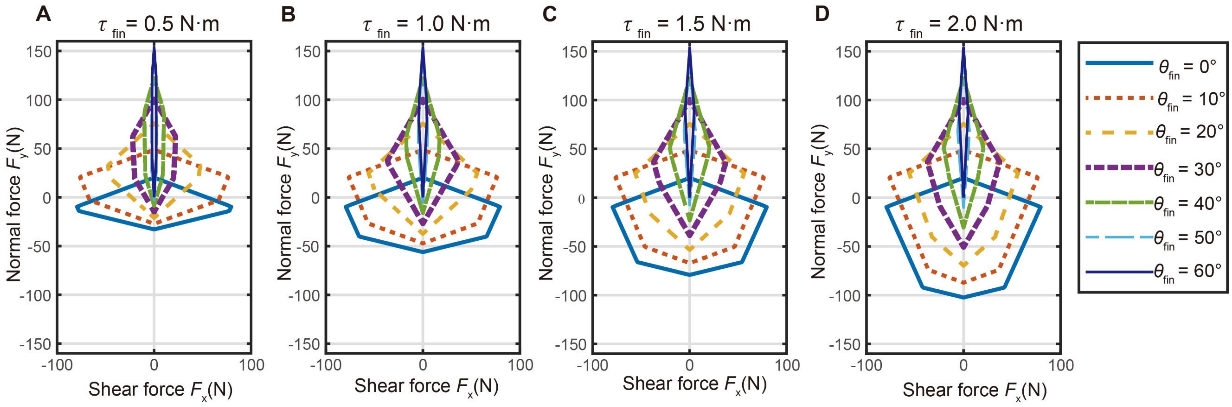

Figure S9. Attachment force generated by the opposed clamping mechanism of SPASAS gripper without footpad. τ_{pha} denotes the driving torque of each finger. γ_{pha} denotes the angle between the phalange and x-axis. **A)** τ_{pha} = 0.5 N·m. **B)** τ_{pha} = 1.0 N·m. **C)** τ_{pha} = 1.5 N·m. **D)** τ_{pha} = 2.0 N·m.

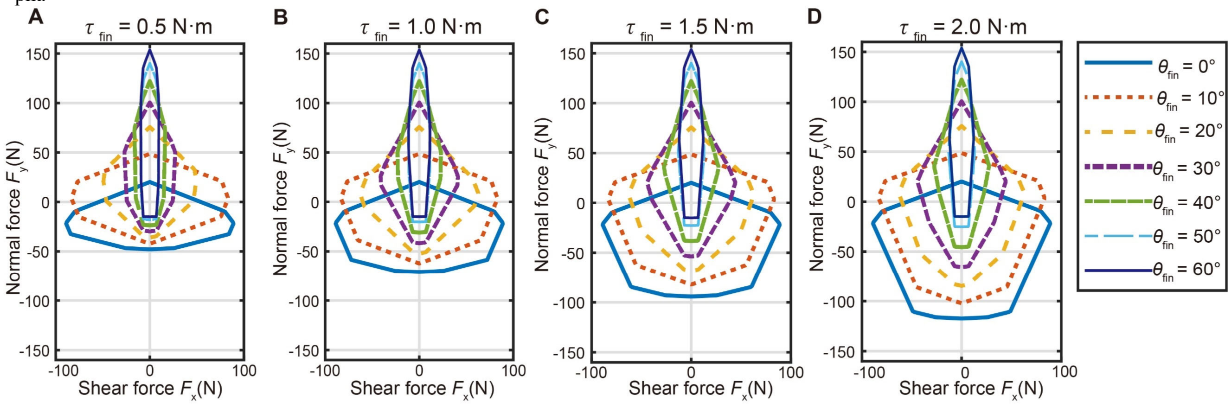

Figure S10. Attachment force generated by the opposed clamping mechanism of SPASAS gripper with footpad. τ_{pha} denotes the driving torque of each finger. γ_{pha} denotes the angle between the phalange and x-axis. **A)** τ_{pha} = 0.5 N·m. **B)** τ_{pha} = 1.0 N·m. **C)** τ_{pha} = 1.5 N·m. **D)** τ_{pha} = 2.0 N·m.

When a soft rubber footpad is added to the bottom of the mechanism, it can provide support force when the opposed clamping mechanism is compressed inward and zero force when pulled outward. Assuming that the surface to be attached can generate a pre-compression force of 0 to 10 N on the footpad during attachment, the resulting attachment force produced by the opposed clamping mechanism can be calculated, as shown in Figure S10. It allows for greater shear attachment force under load than the mechanism without this pad. The load capacity of the opposed clamping mechanism at each location, compared to Figure S9, is significantly larger. Additionally, the extra support provided by the soft footpad can enhance the capacity for normal support of the mechanism.

Based on the above analysis, increasing the contact between the footpad and the rock surface during the attachment process can expand the range of loads that the opposed clamping mechanism can withstand. Therefore, in the actual attachment process, the footpad is pre-compressed first to work in conjunction with the microspines for attachment.

From Figure S9 and Figure S10, it can be found that when γ_{pha} between the fingers and the *x*-axis is small, feasible load region consists of predominantly shear attachment forces, with small normal attractions. As γ_{pha} increases, the maximum normal attraction that opposed clamping mechanism can generate gradually increases, though support and shear attachment force decrease. Therefore, to generate significant tangential attachment force, γ_{pha} should be small. For substantial normal attachment force, γ_{pha} should be larger.

S1.3. Mobility analysis of the robot

The mammal-type leg configuration with $R_rR_yR_y$ joint layout, the sprawling-type leg configuration with $R_pR_yR_y$ joint layout, and the five-bar parallel mechanism leg configuration are used in most of current quadruped robots. Where R denotes the rotation joint, the subscript is the rotation axis direction, and r, p, and y are roll, pitch, and yaw, respectively [1]. The mammal-type leg is under the body while walking. Its advantages include low joint torques, a high payload capacity, and narrow foothold spacing. The support area, on the other hand, is small, and the center of mass (COM) is high. When standing, the sprawling-type leg spreads on both sides of the robot body and swings forward when walking. It has a large support area and a low COM, which are both advantages. However, when standing, the joint torque is large, and the inertia force is large when the legs swing. Parallel-type legs have a high load-bearing capacity, high stiffness, and good dynamic performance, but their workspace is limited.

During climbing, the direction of gravity changes relative to the body, and a large joint torque must be generated regardless of that configuration. The robot body can be closer to the supporting surface due to the sprawling-type leg configuration, resulting in a smaller overturning moment for the attachment device. The mammal-type or parallel-type configuration shifts the COM of the robot away from the supporting surface, making it more easily overturned. Furthermore, the sprawling-type configuration has a larger workspace when extended, allowing the robot to select a more diverse foothold on the supporting surface. The configuration of MARCBot is sprawling-type because free-climbing robots in extreme environments require high stability with relatively low dynamic performance.

When the gripper attaches to the rock surface, a force closure or form closure is generated, so that it can be considered fixed to the rock. A typical legged robot with three-DOF legs can generally form equivalent passive ball-hinges between its feet and the supporting surface. As a result, when the robot walks, the attitudes of its feet is generally inessential. If a typical robot foot is replaced with a spiny gripper, the corresponding attitude will become uncontrollable in a 3-DOF leg. After the gripper grasps a rock, the robot can hardly move freely because the body cannot change its attitude relative to the gripper. Thus, more joints should be added to standard configurations of quadruped robots in order for it to adjust the attitudes of grippers.

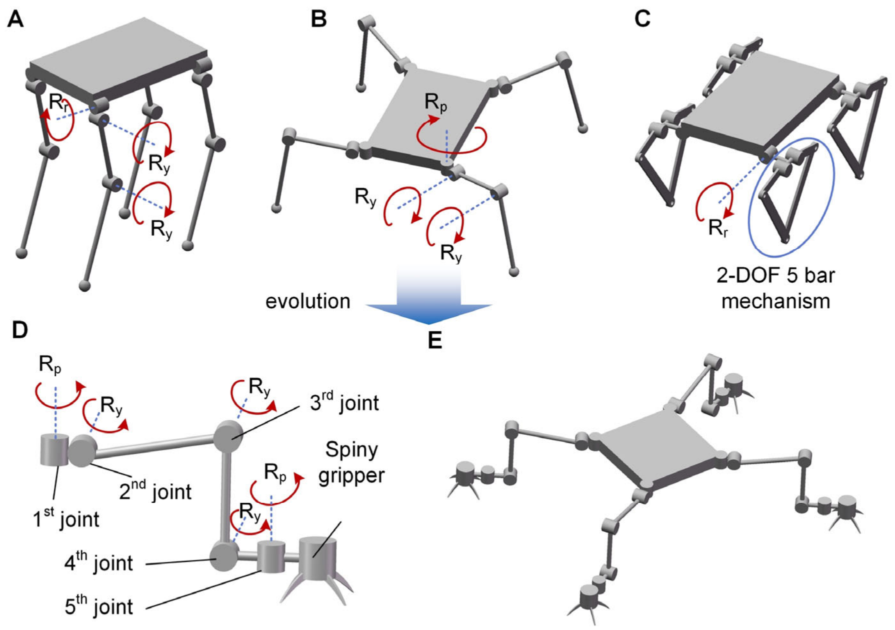

Figure S11. Evolution of the configuration of MARCBot. A) Mammal type. **B**) Sprawl type. **C**) Parallel mechanism type. **D**) The single limb configuration of MARCBot. **E**) Overall configuration of MARCBot.

The fourth joint parallel to the third joint was added to the sprawling-type leg configuration of the previous generation of MARCBot [2] and the climbing robot [3]. This enabled the robot to complete a planar translational motion and move forward in a singular configuration when the axes of the second joint and third joint of stance legs were parallel. When two or more grippers attaching to the surface, the robot body has 3 DOFs (translations along the y and the z-axis, and rotation about the x-axis of frame $\{B\}$ in Figure S4). The robot can only move along the z axes when not in this singularity configuration. However, in a general configuration with the limbs spread, such a leg configuration does not allow the climbing robot to complete the climbing motion, resulting in a limited choice of foothold for the robot.

Here, MARCBot has five-DOF legs with the fifth joint before the gripper is parallel to the first joint as shown in Figrue 5 in the main text. It is offset from the center of the gripper. If the fifth joint is positioned directly above the gripper, the gripper will bear a larger torque while climbing, increasing the possibility of attachment failure.

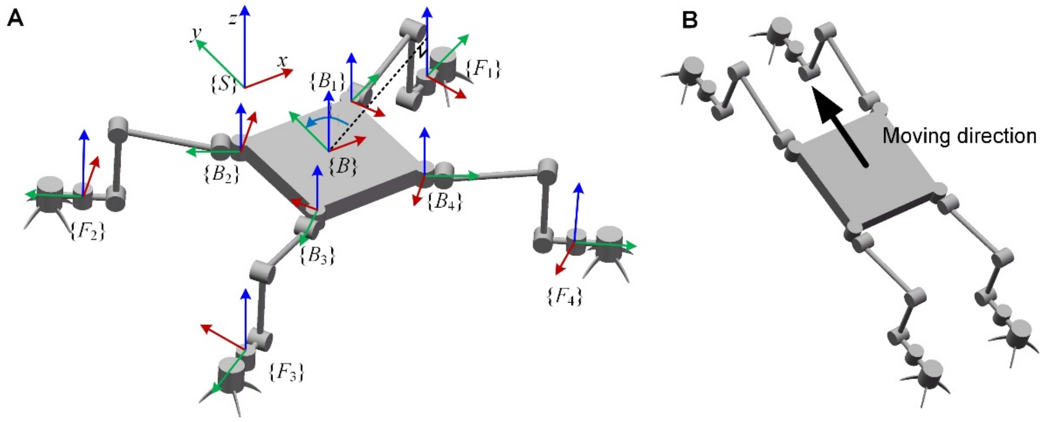

Figure S12. Mobility analysis model. A) The frames for mobility analysis. **B)** Singular configuration.

The mobility of free-climbing robot can be analyzed using the screw theory referred to the method in [3-4]. The motion-screw system of each leg can be established first, and the constraint-screw system of each leg can be produced from the reciprocal screws. The common constraint-screw system of the robot body can be derived by intersecting these constraint-screw systems. As a result, we can obtain the mobility of the robot and determine what movements it is capable of. The twist of each joint can be written as

$$\boldsymbol{s} = \begin{pmatrix} \boldsymbol{\omega} \\ \boldsymbol{r} \times \boldsymbol{\omega} \end{pmatrix} \tag{S1}$$

where $\boldsymbol{\omega} \in \mathbb{R}^3$ denotes the angular velocity of the joint, and $\boldsymbol{r} \in \mathbb{R}^3$ denotes the position vector of the joint in the coordinate system. It is considered that the gripper is always kept parallel to the body during climbing. Take the first leg as an example. In the foot frame of the first leg $\{F_1\}$ as illustrated in Figure S12A, the twists of the five joints in one leg can be written as

$$\begin{cases} \boldsymbol{s}_{11} = \begin{pmatrix} 0 & 0 & 1 & d_1 & e_1 & 0 \end{pmatrix}^{\mathrm{T}} \\ \boldsymbol{s}_{12} = \begin{pmatrix} \cos\theta_{1,5} & \sin\theta_{1,5} & 0 & d_2 & e_2 & f_2 \end{pmatrix}^{\mathrm{T}} \\ \boldsymbol{s}_{13} = \begin{pmatrix} \cos\theta_{1,5} & \sin\theta_{1,5} & 0 & d_3 & e_3 & f_3 \end{pmatrix}^{\mathrm{T}} \\ \boldsymbol{s}_{14} = \begin{pmatrix} \cos\theta_{1,5} & \sin\theta_{1,5} & 0 & 0 & 0 & f_4 \end{pmatrix}^{\mathrm{T}} \\ \boldsymbol{s}_{15} = \begin{pmatrix} 0 & 0 & 1 & 0 & 0 & 0 \end{pmatrix}^{\mathrm{T}} \end{cases} \tag{S2}$$

where $\theta_{1,5}$ denotes the joint variable of the fifth joint in the first leg, and the other scalar quantities (including d_i, e_i and f_i) can be represented by the lengths of leg segments and joint variables. The elements of this motion screw system are all linearly independent of one another. Because the reciprocal product of their reciprocal screw for each element is **0**, the reciprocal screw of them is defined as

$$\boldsymbol{s}_{11}^{r} = \begin{pmatrix} 0 & 0 & 0 & -\sin\theta_{1,5} & \cos\theta_{1,5} & 0 \end{pmatrix}^{\mathrm{T}} \tag{S3}$$

The physical meaning of Equation (S3) is a constraint moment along the intersectant axis of the leg plane and the *xy* plane. It means that the rotation along this axis is constrained in this configuration. Establish the space frame $\{S\}$ whose axis parallel to each axis of the body coordinate system $\{B\}$ as illustrated in Figure S12A. The transformation matrix from $\{F_1\}$ to $\{S\}$ can be written as

$$\boldsymbol{T}_{F_1}^{S} = \begin{pmatrix} \boldsymbol{R}_{F_1}^{S} & \boldsymbol{t}_{F_1}^{S} \\ \boldsymbol{0} & 1 \end{pmatrix} = \begin{pmatrix} \cos\alpha_1 & -\sin\alpha_1 & 0 & a_1 \\ \sin\alpha_1 & \cos\alpha_1 & 0 & b_1 \\ 0 & 0 & 1 & c_1 \\ 0 & 0 & 0 & 1 \end{pmatrix} \tag{S4}$$

where $\boldsymbol{R}_{F_1}^{S} \in SO(3)$ is the rotation matrix that transforms the $\{F_1\}$ to $\{S\}$, $\boldsymbol{t}_{F_1}^{S} \in \mathbb{R}^3$ is the translation matrix from $\{F_1\}$ to $\{S\}$, and a_1, b_1, c_1 are the position parameters of the first foot. Thus, the constrained screw in $\{S\}$ can be written as

$$\begin{aligned} \boldsymbol{s}_{S_1}^{\mathrm{r}} &= \mathrm{Ad}\left(\boldsymbol{T}_{F_1}^{S}\right)\boldsymbol{s}_{11}^{\mathrm{r}} = \begin{pmatrix} \boldsymbol{R}_{F_1}^{S} & \boldsymbol{0} \\ \hat{\boldsymbol{t}}_{F_1}^{S}\boldsymbol{R}_{F_1}^{S} & \boldsymbol{R}_{F_1}^{S} \end{pmatrix}\boldsymbol{s}_{11}^{\mathrm{r}} \\ &= \begin{pmatrix} \boldsymbol{0}_{1\times3} & -c\alpha_1 s\theta_{15} - s\alpha_1 c\theta_{15} & -s\alpha_1 s\theta_{15} + c\alpha_1 c\theta_{15} & 0 \end{pmatrix}^{\mathrm{T}} \end{aligned} \tag{S5}$$

where $\mathrm{Ad}\left(\boldsymbol{T}_{F_1}^{S}\right)$ means the adjoint representation of $\boldsymbol{T}_{F_1}^{S}$, and the detailed mathematical expression of $\mathrm{Ad}(\cdot)$ can be found in Foundation of mathematics. The constraint screws of other legs can be derived in a similar form as

$$\begin{aligned} \boldsymbol{s}_{S2}^{\mathrm{r}} &= \begin{pmatrix} \boldsymbol{0}_{1\times3} & -c\alpha_2 s\theta_{2,5} - s\alpha_2 c\theta_{2,5} & -s\alpha_2 s\theta_{2,5} + c\alpha_2 c\theta_{2,5} & 0 \end{pmatrix}^{\mathrm{T}} \\ \boldsymbol{s}_{S3}^{\mathrm{r}} &= \begin{pmatrix} \boldsymbol{0}_{1\times3} & -c\alpha_3 s\theta_{3,5} - s\alpha_3 c\theta_{3,5} & -s\alpha_3 s\theta_{3,5} + c\alpha_3 c\theta_{3,5} & 0 \end{pmatrix}^{\mathrm{T}} \\ \boldsymbol{s}_{S4}^{\mathrm{r}} &= \begin{pmatrix} \boldsymbol{0}_{1\times3} & -c\alpha_4 s\theta_{4,5} - s\alpha_4 c\theta_{4,5} & -s\alpha_4 s\theta_{4,5} + c\alpha_4 c\theta_{4,5} & 0 \end{pmatrix}^{\mathrm{T}} \end{aligned} \tag{S6}$$

The fourth and fifth elements of each constraint screw are linearly independent in a general configuration. The constraint-screw system of the robot body $\boldsymbol{S}_{B}^{\mathrm{r}}$ is the union of constraint-screw of the stance legs. The robot will be in an over-constrained state with at bipedal, tripedal, and quadrupedal supports. A base set of $\boldsymbol{S}_{B}^{r}$ can be written as

$$\begin{cases} \boldsymbol{s}_{B1}^{r} = \begin{pmatrix} 0 & 0 & 0 & 1 & 0 & 0 \end{pmatrix}^{\mathrm{T}} \\ \boldsymbol{s}_{B2}^{r} = \begin{pmatrix} 0 & 0 & 0 & 0 & 1 & 0 \end{pmatrix}^{\mathrm{T}} \end{cases} \tag{S7}$$

The physical meaning of Equation (S7) indicates that the rotation of the robot body along the *x*- and y-axes is constrained. In a default configuration, the robot has 4 DOF and can move translationally and rotate along the z-axis. As a result, while climbing, the robot can move its body in any direction and change its yaw angle. When the robot is in the special configuration as shown in Figure S12B. that the planes of the legs are parallel to each other, that α_1=α_3=0, α_2=$\alpha_4 = \pi$, the only constraint-screw of the robot body is

$$\boldsymbol{s}_{B}^{r} = \begin{pmatrix} 0 & 0 & 0 & 0 & 1 & 0 \end{pmatrix}^{\mathrm{T}} \tag{S8}$$

The foregoing derivation method demonstrates that the robot is only limited to rotation along the y-axis in this special configuration, whereas can achieve translation in space and rotation along the x-axis and z-axis. The assessment of body mobility shows that the MARCBot design, together with the great adaptable capacity of the SPASAS gripper, can meet the needs of free climbing. This robot configuration has the fewest number of DOFs to execute such a maneuver on an inclined terrain. If we continue to add the active DOF of the robot legs, the robot movement will become more flexible. However, this would increase the overall mass of the legs and the robot, as well as the load on the SPASAS gripper. The dynamic performance of robot is weakened when walking on flat ground, making all-terrain adaption more challenging. Furthermore, because attachment devices are frequently large in such climbing robots, the redundant DOF may make workspace design and motion planning difficult.

S1.4. Kinematics model of the robot

The forward kinematics (FK) of MARCBot on $SE(3)$ are modeled using the product of exponential (PoE) formula [6]. The geometric method is used to solve the inverse kinematics problem of the robot. Because of the constraint feature between the spiny gripper and the substrate, different inverse kinematics solutions are used in the stance and swing phases for the proposed five-DOF limb.

S1.3.1. Forward Kinematics (FK).

Brokett proposed the FK modeling approach based on PoE formula in 1984 [6]. The Cartesian coordinate system of the leg is first established for the forward kinematics of the swing leg, with the coordinate system of the first joint of the ith leg $\{B_i\}$ as the leg coordinate system. The unit twists of the jth joint of the ith leg in the initial configuration in $\{B_i\}$ can be expressed as

$$\begin{cases} \boldsymbol{s}_1 = \begin{pmatrix} 0 & 0 & 1 & 0 & 0 & 0 \end{pmatrix}^{\mathrm{T}} \\ \boldsymbol{s}_2 = \begin{pmatrix} 1 & 0 & 0 & 0 & 0 & -l_1 \end{pmatrix}^{\mathrm{T}} \\ \boldsymbol{s}_3 = \begin{pmatrix} 1 & 0 & 0 & 0 & 0 & -l_1 - l_2 \end{pmatrix}^{\mathrm{T}} \\ \boldsymbol{s}_4 = \begin{pmatrix} 1 & 0 & 0 & 0 & 0 & -l_1 - l_2 - l_3 \end{pmatrix}^{\mathrm{T}} \\ \boldsymbol{s}_5 = \begin{pmatrix} 0 & 0 & 1 & 0 & 0 & -l_1 - l_2 - l_3 - l_4 \end{pmatrix}^{\mathrm{T}} \end{cases} \tag{S9}$$

where l_1 to l_4 and h_5 are the length parameters of the segments of the robot leg, as shown in Figure S13. Then the PoE FK formula of the single leg can be written as

$$\boldsymbol{T}_{Fi}^{B} = {}_{Bi}^{B}\boldsymbol{T} e^{\theta_{i,1}\hat{s}_{i,1}} e^{\theta_{i,2}\hat{s}_{i,2}} e^{\theta_{i,3}\hat{s}_{i,3}} e^{\theta_{i,4}\hat{s}_{i,4}} e^{\theta_{i,5}\hat{s}_{i,5}}\, {}^{0}\boldsymbol{T}_{Fi}^{Bi} \tag{S10}$$

where $\theta_{i,j}$ denotes the variable of the jth joint of the ith leg, $\boldsymbol{T}_{Bi}^{B}$ denotes the transformation matrix from $\{B_i\}$ to $\{B\}$, ${}^{0}\boldsymbol{T}_{Fi}^{Bi}$ denotes the initial configuration of the ith leg in $\{B_i\}$ and $e^{\theta_{i,j}\hat{s}_{i,j}} \in SE(3)$ whose detailed expression can be found in Foundation of mathematics.

S1.3.2. Inverse Kinematics (IK).

Because the robot only has five DOFs in one lFimb, whereas spatial rigid body motion has 6 DOF in the configuration space, the motion of the limb alone cannot guarantee simultaneous satisfaction for a given desired position and attitude.Consequensely, the robot employs various inverse kinematic solutions during the stance and swing phases. During the swing phase, the joint variables of the fourth and fifth joints are constant, while the first three joints are controlled to make the gripper move to desired positions. This ensures that the attachment device is positioned correctly at the top of the foothold. After the SPASAS gripper has been attached to the rock, the limb will enter the stance phase. At this point, the fifth joint coordinate system is considered a tool coordinate system, and its configuration in $\{S\}$ will not change during this

phase. The function of fourth and fifth joints is to control the attitude of the gripper. IK can be solved using the geometric method and the corresponding geometric model is illustrated as Figure S13. Let the configuration of the robot in the space frame {S} be $\boldsymbol{T}$, the planned trajectory each gripper in {S} be $\boldsymbol{T}_{Fi}^{S}$. The configuration of the gripper in {B} can be written as

$$\boldsymbol{T}_{Fi}^{B} = \boldsymbol{T}^{-1}\boldsymbol{T}_{Fi}^{S} = \begin{pmatrix} \boldsymbol{R}_{Fi} & \boldsymbol{P}_{Fi} \\ \boldsymbol{0} & 1 \end{pmatrix} \tag{S11}$$

where $\boldsymbol{P}_{Fi} = \begin{pmatrix} p_{Fix} & p_{Fiy} & p_{Fiz} \end{pmatrix} \in \mathbb{R}^3$ denotes the position vector of the gripper in {B_i}. l_{xy} illustrated in Figure S13B can be obtained

$$l_{xy} = \left({p_{Fix}}^2 + {p_{Fiy}}^2 \right)^{\frac{1}{2}} \tag{S12}$$

In swing phase, the joint variable of the fifth joint is given as a value such that $\theta_{i,5} = \pm\alpha$, which is predetermined. The sine theorem can be used to calculate β in Figure S13.

$$\beta = \arcsin\left(\frac{\sin\frac{5\pi}{6}}{l_{xy}'} l_5 \right) \tag{S13}$$

where $l_{xy}' = \left({l_{xy}}^2 + l_5^2 - 2l_{xy}l_5\cos(\alpha-\beta) \right)^{\frac{1}{2}}$. Other joint variables for each joint can be obtained using trigonometric functions based on the geometric relationship of the parameters in Figure S13:

$$\boldsymbol{\theta}_i = \begin{pmatrix} \theta_{i,1} \\ \theta_{i,2} \\ \theta_{i,3} \\ \theta_{i,4} \\ \theta_{i,5} \end{pmatrix} = \begin{pmatrix} \operatorname{arccot}\frac{p_{Fix}}{p_{Fiy}} - \frac{\pi}{2} - \alpha \\ \arccos\frac{l_2^2 + l_8^2 - l_3^2}{2l_2l_8} + \arctan\frac{l_7}{l_6} \\ \arccos\frac{l_2^2 - l_8^2 + l_3^2}{2l_2l_3} - \pi \\ -\theta_{i,3} - \theta_{i,2} \\ \pm\alpha \end{pmatrix} \tag{S14}$$

where $\begin{cases} l_6 = l_{xy}' - (l_1 + l_4) \\ l_7 = p_{F_iz} + h_5 \\ l_8 = \sqrt{l_6^2 + l_7^2} \end{cases}$.

When the gripper grasping rocks, the first and fifth joint variables are noted as $\theta_{i,1}(0)$ and $\theta_{i,5}(0)$ respectively. The attitude and position of the gripper do not change during the stance phase. The position of the fifth joint is

$$\begin{pmatrix} p_{Fix}' \\ p_{Fiy}' \\ p_{Fiz}' \end{pmatrix} = \begin{pmatrix} p_{Fix} + \cos\left(\frac{\pi}{2} + \theta_{i,1}(0) + \theta_{i,5}(0) l_5 \right) \\ p_{Fiy} - \sin\left(\frac{\pi}{2} + \theta_{i,1}(0) + \theta_{i,5}(0) \right) l_5 \\ p_{Fiz} + h_5 \end{pmatrix} \tag{S15}$$

Thus $\theta_{i,1}$ can be obtained as follows:

$$\theta_{i,1} = \operatorname{atan2}\left(p_{Fy}', p_{Fx}'\right) - \frac{\pi}{2} \tag{S16}$$

Then $\theta_{i,5}$ can be get as follows:

$$\theta_{i,5} = \theta_{i,1}(0) + \theta_{i,5}(0) - \theta_{i,1} \tag{S17}$$

where $\theta_{i,1}(0)$ and $\theta_{i,5}(0)$ denote the initial joint variable at the start of stance phase. The expressions of other joint variables are the same as the IK solutions of (S14) at the swing phase.

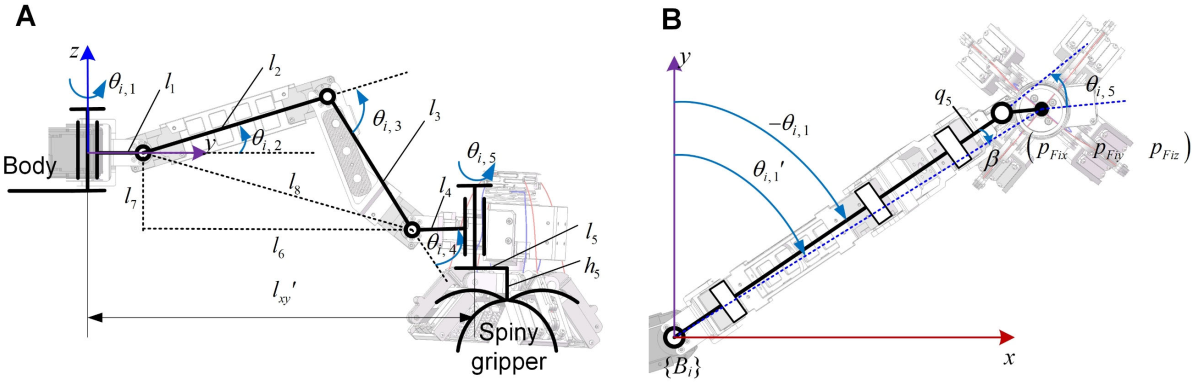

Figure S13. Geometric model for IK solution. **A**) View in *yz*-plane. **B**) View in *xy*-plane.

S1.5. Simplified dynamics model of the robot

The dynamic equation of the robot body is written as

$$\frac{\mathrm{d}}{\mathrm{d}t}(\boldsymbol{M}_B \boldsymbol{V}_B) = \boldsymbol{G}_B + \sum \boldsymbol{F}_{i,1} \tag{S18}$$

where $\boldsymbol{M}_B \in \mathbb{R}^{6\times6}$ denotes the spatial inertia matrix of the robot body in $\{S\}$, $\boldsymbol{V}_B \in \mathbb{R}^6$ denotes the generalized velocity of the robot, $\boldsymbol{G}_B \in \mathbb{R}^6$ denotes the gravitational wrench on the body, and $\boldsymbol{F}_{i,1} \in \mathbb{R}^6$ denotes the first joint wrench of the ith leg. The spatial inertial matrix of the single rigid body is defined as

$$\boldsymbol{M}_B = \left(\mathrm{Ad}(\boldsymbol{T})\right)^{\mathrm{T}} \boldsymbol{M}_B^B \mathrm{Ad}(\boldsymbol{T}) = \left(\mathrm{Ad}(\boldsymbol{T})\right)^{\mathrm{T}} \begin{bmatrix} \overline{\boldsymbol{I}}_B & 0 \\ 0 & m_B \boldsymbol{I}_{3\times3} \end{bmatrix} \mathrm{Ad}(\boldsymbol{T}) \tag{S19}$$

where $\boldsymbol{M}_B^B \in \mathbb{R}^{6\times6}$ is the inertial matrix in $\{B\}$, $\mathrm{Ad}(\boldsymbol{T}) \in \mathbb{R}^{6\times6}$ is the adjoint represent of $\boldsymbol{T}$, $\overline{\boldsymbol{I}}_B \in \mathbb{R}^{3\times3}$ is the rotational inertial matrix in $\{B\}$, m_B is the mass of the body, and $\boldsymbol{I}_{3\times3}$ is the unit diagonal matrix. The left side of (S18) can be written as the centroid dynamics form as

$$\frac{\mathrm{d}}{\mathrm{d}t}(\boldsymbol{M}_B \boldsymbol{V}) = \boldsymbol{M}_B \dot{\boldsymbol{V}} - \mathrm{ad}(\boldsymbol{V})^{\mathrm{T}} \boldsymbol{M}_B \boldsymbol{V} \approx \boldsymbol{M}_B \dot{\boldsymbol{V}} \tag{S20}$$

where $\mathrm{ad}(\boldsymbol{V})$ is the adjoint represent of $\boldsymbol{V}$, whose expression can be found in Foundation of mathematics. This approximation, which ignores the effect of the precession and nutation of the rotating body [7].

Because the legs move slowly during climbing, the inertial force, centripetal force, and Coriolis force are all ignored, and only the gravity term is retained in its dynamics formula. In supporting legs, let $\boldsymbol{F}_{i,\text{con}}^{i,\text{end}} = \begin{pmatrix} \tau_{ix} & \tau_{iy} & \tau_{iz} & f_{ix} & f_{iy} & f_{iz} \end{pmatrix}^{\mathrm{T}} \in \mathbb{R}^6$ denote the contact wrench acting on the gripper at the ith leg. Then, for the fifth joint,

$$\mathrm{Ad}\left(\boldsymbol{T}_{i,5}^{i,6}\right)^{\mathrm{T}} \boldsymbol{F}_{i,\text{con}}^{i,\text{end}} + \boldsymbol{F}_{i,5}^{i,5} + \boldsymbol{G}_{i,5}^{i,5} = \boldsymbol{0} \tag{S21}$$

where $\mathrm{Ad}\left(\boldsymbol{T}_{i,5}^{i,6}\right) \in \mathbb{R}^{6\times6}$ denotes the adjoint map of $\boldsymbol{T}_{i,5}^{i,6}$, which can transform $\boldsymbol{F}_{i,\text{con}}^{i,\text{end}}$ into the fifth joint coordinate, $\boldsymbol{F}_{i,5}^{i,5}$ denotes the wrench exerting on the fifth joint, and $\boldsymbol{G}_{i,5}^{i,5}$ denote the gravity wrench in the fifth joint coordinate. Thus, $\boldsymbol{F}_{i,5}^{i,5}$ can be found as follows:

$$\boldsymbol{F}_{i,5}^{i,5} = -\mathrm{Ad}\left(\boldsymbol{T}_{i,5}^{i,\mathrm{end}}\right)^{\mathrm{T}} \boldsymbol{F}_{i,\mathrm{con}}^{i,\mathrm{end}} - \boldsymbol{G}_{i,5}^{i,5} \tag{S22}$$

Iterate the joint wrench inward, and the wrench at the kth joint $\boldsymbol{F}_{i,k}^{i,k}$ can be derived as follows:

$$\boldsymbol{F}_{i,k}^{i,k} = -{}^{i,k}\boldsymbol{G}_{i,k} - \sum_{j=k+1}^{5} \mathrm{Ad}\left(\boldsymbol{T}_{i,k}^{i,j}\right)^{\mathrm{T}} \boldsymbol{G}_{i,j}^{i,j} - \mathrm{Ad}\left(\boldsymbol{T}_{i,5}^{i,\mathrm{end}}\right)^{\mathrm{T}} \boldsymbol{F}_{i,\mathrm{con}}^{i,\mathrm{end}} \tag{S23}$$

To get the joint torques, we can pair $\boldsymbol{F}_{i,k}^{i,k}$ with the joint screws. The joint torque $\tau_{i,k}$ can be derived as follows:

$$\tau_{i,k} = \left(\boldsymbol{F}_{i,k}^{i,k}\right)^{\mathrm{T}} \boldsymbol{s}_{i,k}^{i,k} \tag{S24}$$

where $\boldsymbol{s}_{i,k}^{i,k}$ is the twist in the kth joint coordinate of the ith leg. The joint wrenches in a swing leg have a similar form with Equation (S23), but it is not subject to external constraint wrench at its gripper. Thus, $\boldsymbol{F}_{i,\mathrm{con}}^{i,\mathrm{end}} = \boldsymbol{0}$ in a swing leg. As a result, the swing leg joint can be calculated as

$${}^{i,k}\boldsymbol{F}_{i,k} = -\sum_{j=k+1}^{5} \boldsymbol{G}_{i,j}^{i,k} = -\boldsymbol{G}_{i,k}^{i,k} - \sum_{j=k+1}^{5} \mathrm{Ad}\left(\boldsymbol{T}_{i,k}^{i,j}\right)^{\mathrm{T}} \boldsymbol{G}_{i,j}^{i,k} \tag{S25}$$

Take Equation (S20), Equation (S23) and Equation (S25) into Equation (S18),

$$\boldsymbol{M}_B\dot{\boldsymbol{V}}_B = \boldsymbol{G}_B + \sum_{i=1}^{4} \mathrm{Ad}\left(\boldsymbol{T}_{S}^{i,1}\right)^{\mathrm{T}} \underbrace{\left(\boldsymbol{G}_{i,1}^{i,1} + \sum_{j=2}^{5} \mathrm{Ad}\left(\boldsymbol{T}_{i,1}^{i,j}\right)^{\mathrm{T}} \boldsymbol{G}_{i,j}^{i,j}\right)}_{\boldsymbol{G}_i} + \sum_{i=1}^{n} \mathrm{Ad}\left(\boldsymbol{T}_{S}^{i,6}\right)^{\mathrm{T}} \boldsymbol{F}_{i,\mathrm{con}}^{i,\mathrm{end}} \tag{S26}$$

where $n \in \mathbb{N}^+$ represents the number of supporting legs. All the attachment wrenches from legs can be rewrite as follows:

$$\boldsymbol{F} = \left(\left(\boldsymbol{F}_{1,\mathrm{con}}^{1,\mathrm{end}}\right)^{\mathrm{T}} \quad \left(\boldsymbol{F}_{2,\mathrm{con}}^{2,\mathrm{end}}\right)^{\mathrm{T}} \quad \left(\boldsymbol{F}_{3,\mathrm{con}}^{3,\mathrm{end}}\right)^{\mathrm{T}} \quad \left(\boldsymbol{F}_{4,\mathrm{con}}^{4,\mathrm{end}}\right)^{\mathrm{T}}\right) \tag{S27}$$

The Equation (S26) can be rewritten as follows:

$$\boldsymbol{A}\boldsymbol{F} - \boldsymbol{B} = \boldsymbol{0} \tag{S28}$$

where $\boldsymbol{A} = \left(\mathrm{Ad}\left(\boldsymbol{T}_{S}^{1,6}\right)^{\mathrm{T}} \quad \cdots \quad \mathrm{Ad}\left(\boldsymbol{T}_{S}^{n,6}\right)^{\mathrm{T}}\right) \in \mathbb{R}^{6\times 6n}$,

$$\boldsymbol{B} = \boldsymbol{M}_B\dot{\boldsymbol{V}}_B - \boldsymbol{G}_B - \sum_{i=1}^{4}\left(\mathrm{Ad}\left(\boldsymbol{T}_{S}^{i,1}\right)^{\mathrm{T}} \boldsymbol{G}_{i,1}^{i,1} + \sum_{j=2}^{5} \mathrm{Ad}\left(\boldsymbol{T}_{S}^{i,j}\right)^{\mathrm{T}} \boldsymbol{G}_{i,j}^{i,j}\right).$$

The Equation (S28) is used in the QP-based balance controller for the optimal distribution of attachment wrenches.

S1.6. Body tracking PD controller

The desired and actual states of the fuselage are the input to the fuselage controller, whereas the output is the required external virtual force of the fuselage, which is also the input to the support leg control. The goal is to make the actual motion trajectory $\boldsymbol{T}$ and actual general spatial velocity $\boldsymbol{V}$ track the desired trajectory $\boldsymbol{T}_{\mathrm{d}}$ and desired general spatial velocity $\boldsymbol{V}_{\mathrm{d}}$. Controlling the wrench exerted by each leg on the robot's body allowed the robot to track the desired general spatial acceleration $\dot{\boldsymbol{V}}_{\mathrm{d}}$. Therefore, the generated reference general spatial acceleration $\dot{\boldsymbol{V}}_{\mathrm{ref}}$ was used as the output of the fuselage trajectory tracking controller, which was translated to the input of the force control loop. A common method is to define the motion error in $\mathbb{R}^3$ and $SO(3)$ and then control the translation and rotation separately according to the motion error [8]. PD control law was applied as a body tracking controller; the details are provided in Supplementary Materials. The output of the body trajectory tracking controller was obtained. Subsequently, the reference wrench required by the support leg to converge the body trajectory to the desired value was determined.

The position error is defined as $\tilde{\boldsymbol{P}} = \boldsymbol{P}_{\mathrm{d}} - \boldsymbol{P}$, where $\boldsymbol{P}_{\mathrm{d}} \in \mathbb{R}^3$ is the desired position of the robot and $\boldsymbol{P}_B \in \mathbb{R}^3$ is the practical position of the robot. The linear velocity error of the robot is

defined as $\tilde{\boldsymbol{v}} = \boldsymbol{v}_{\mathrm{d}} - \boldsymbol{v}$, where $\boldsymbol{v}_{\mathrm{d}} \in \mathbb{R}^3$ denotes the desired linear velocity of the robot body and $\boldsymbol{v} \in \mathbb{R}^3$ represents practical linear velocity of the robot body. The PD control law is used to calculate the reference line acceleration of the body controller output as

$$\dot{\boldsymbol{v}}_{\mathrm{ref}} = \boldsymbol{K}_{\mathrm{p1}}\tilde{\boldsymbol{P}} + \boldsymbol{K}_{\mathrm{d1}}\tilde{\boldsymbol{v}} \tag{S29}$$

where $\boldsymbol{K}_{\mathrm{p1}} \in \mathbb{R}^{3\times3}$ denotes the proportional coefficient matrix and $\boldsymbol{K}_{\mathrm{d1}} \in \mathbb{R}^{3\times3}$ denotes the differential coefficient matrix.

The attitude of the robot cannot be decoupled into mutually uncorrelated components along the x, y, and z axes. The error in angular velocity can be defined as $\tilde{\boldsymbol{\omega}} = \boldsymbol{\omega}_{\mathrm{d}} - \boldsymbol{\omega}$,where $\boldsymbol{\omega}_{\mathrm{d}} \in \mathbb{R}^3$ is the desired angular velocity of the body and, and $\boldsymbol{\omega}_B \in \mathbb{R}^3$ is practical angular velocity of the body respectively. $\boldsymbol{R}_{\mathrm{e}} \in \mathbb{R}^{3\times3}$ is defined as rotation error of the robot as

$$\boldsymbol{R}_{\mathrm{e}} = \boldsymbol{R}_{\mathrm{d}}\boldsymbol{R}^{-1} = \boldsymbol{R}_{\mathrm{d}}\boldsymbol{R}^{\mathrm{T}} \tag{S30}$$

where $\boldsymbol{R}_{\mathrm{d}} \in \mathbb{R}^{3\times3}$ is the desired attitude matrix of the robot and $\boldsymbol{R} \in \mathbb{R}^{3\times3}$ denotes the practical attitude matrix of the robot. Use $\boldsymbol{\xi} \in \mathbb{R}^{3\times1}$ to represent the exponential coordinates of $\boldsymbol{R}_{\mathrm{e}} \in \mathbb{R}^{3\times3}$, and the reference angular accelerate can be obtained by PD control law as follows,

$$\dot{\boldsymbol{\omega}}_{\mathrm{ref}} = k_{\mathrm{p2}}\boldsymbol{\xi} + \boldsymbol{K}_{\mathrm{d2}}\tilde{\boldsymbol{\omega}} \tag{S31}$$

where $k_{\mathrm{p2}} \in \mathbb{R}$ is the proportional coefficient and $\boldsymbol{K}_{\mathrm{d2}} \in \mathbb{R}^{3\times3}$ is the differential coefficient matrix.

The output of the body trajectory tracking controller has been available. Then the reference wrench required by the support leg to make the body trajectory converge to the desired value can be calculated.

S1.7. Methods of control groups in climbing tests

S1.7.1 Closed-Loop Inverse Kinematics Control

In climbing robots that utilize high reduction ratios and have lower dynamic requirements, the Closed-Loop Inverse Kinematics (CLIK) control is a fundamental and commonly used approach. Consequently, it is selected as primary comparison object. When using CLIK control, the control diagram is shown in the Figure S14.

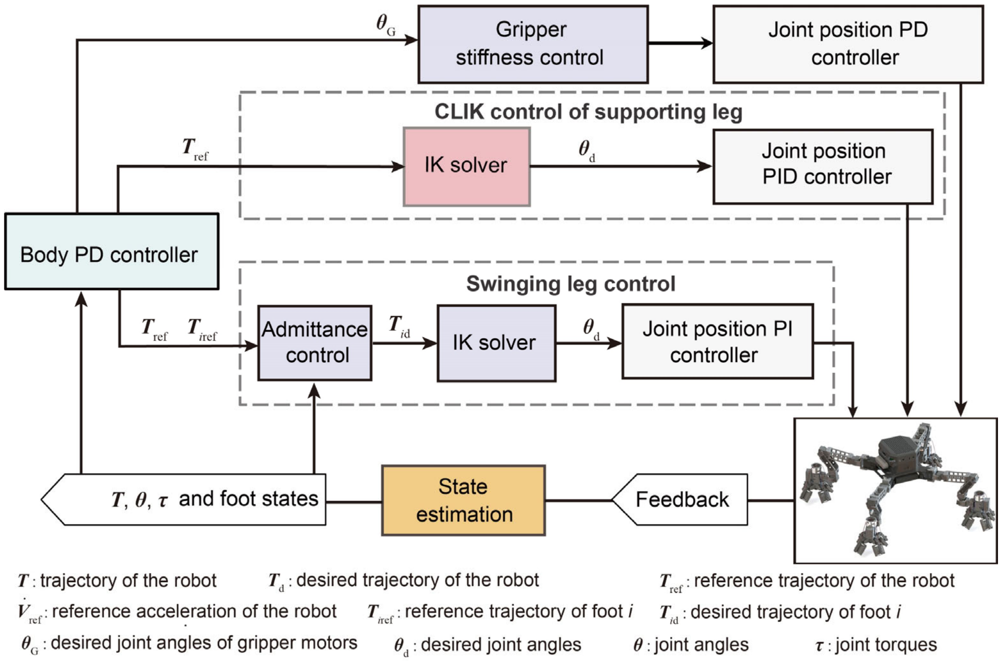

Figure S14. Control block diagram of CLIK control. The different part from the proposed Q-WBC is the supporting leg control loop.

The control of the swinging leg and gripper is the same as the Q-WBC proposed in the main text. The main difference lies in the control of the swinging leg. A PD controller is used to generated the reference trajectory for the robot based on the location and velocity errors. In the swinging leg, this reference trajectory is used with the IK module to solve in real time the required joint angles for the robot's joints. This control does not involve the allocation and calculation of forces.

S1.7.2 Virtual Model Control

The core idea of Virtual Model Control (VMC) is to use imaginary virtual components to connect the robot and the external environment, thereby generating corresponding virtual forces $\boldsymbol{f}_{\mathrm{VM}i}$ to "drive" the robot to achieve the desired motion. The virtual components between MARCBot and environment are shown in Figure S15, that displays only one support leg's virtual spring-damper component; the other support legs, not displayed, use the same virtual model. The VMC control method, as shown in Figure S16, involves support leg control that differs from the method proposed in this article.

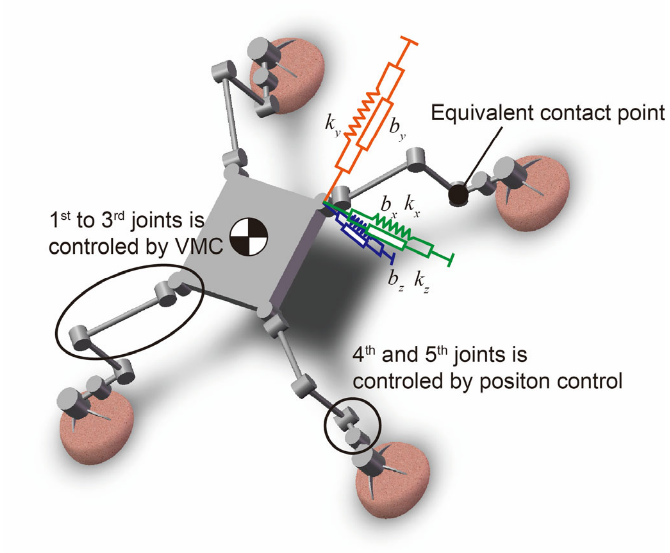

Figure S15. Virtual model of the robot limb.

Here, quadratic programming (QP) is also used to distribute the forces $\boldsymbol{f}_{\mathrm{QP}i}$ from each foot for executing the planned motion. The desired contact forces $\boldsymbol{f}_{i\mathrm{d}}$ at the ith foot are the combined forces of $\boldsymbol{f}_{\mathrm{QP}i}$ and the virtual force $\boldsymbol{f}_{\mathrm{VM}i}$. These virtual forces and Jacobian $\boldsymbol{J}$ are used to obtained the desired joint torques as input for joint control, driving the robot's motion to produce effects similar to those of the virtual components. Since the MARCBot's single leg has five degrees of freedom, it cannot fully control the torques generated at the foot end. Therefore, it is necessary to discard the torque components. Position control is used to drive the servos at the fouth and fifith joints. The position of fouth joint is considered an equivalent contact point with the ground, controlled by the first three joints to achieve the desired contact force at this point. The desired contact force $\boldsymbol{f}_{i\mathrm{d}}$ at the equivalent contact point can be expressed as follows:

$$\boldsymbol{f}_{i\mathrm{d}} = \boldsymbol{f}_{\mathrm{QP}i} + \boldsymbol{f}_{\mathrm{VM}i} \tag{S32}$$

where the virtual force $\boldsymbol{f}_{\mathrm{VM}i}$ generated by the spring-damper virtual components can be expressed as follows:

$$\boldsymbol{f}_{\mathrm{VM}i}=\boldsymbol{K}_{\mathrm{VM}}(\boldsymbol{p}_{i\mathrm{d}}-\boldsymbol{p}_i)+\boldsymbol{B}_{\mathrm{VM}}(\dot{\boldsymbol{p}}_{i\mathrm{d}}-\dot{\boldsymbol{p}}_i)=\begin{bmatrix}k_x&0&0\\0&k_y&0\\0&0&k_z\end{bmatrix}\begin{bmatrix}e_x\\e_y\\e_z\end{bmatrix}+\begin{bmatrix}b_x&0&0\\0&b_y&0\\0&0&b_z\end{bmatrix}\begin{bmatrix}\dot{e}_x\\\dot{e}_y\\\dot{e}_z\end{bmatrix} \quad \text{(S33)}$$

The virtual force $\boldsymbol{f}_{\mathrm{VM}i}$ generated by the virtual components depends on the stiffness and damping coefficient matrices $\boldsymbol{K}_{\mathrm{VM}}$ and $\boldsymbol{B}_{\mathrm{VM}}$, respectively, and the position $\boldsymbol{p}_i$ of foot i with $\boldsymbol{p}_{i\mathrm{d}}$ indicating the desired position, e representing the positional error, and $\dot{e}$ indicating the velocity error, with subscripts denoting their respective directions. Here, $k_x=k_y=k_z$=1000 N/m and $b_x=b_y=b_z$=50 Ns/m. With this setup, the stiffness of the leg is relatively low to ensure compliance during the climbing process, thereby reducing the internal forces. It also ensures that the virtual forces generated remain within the servo's driving capability. The relationship between the virtual forces generated by the virtual components and the desired joint torques is as follows:

$$\boldsymbol{\tau}=\boldsymbol{J}^{\mathrm{T}}\boldsymbol{f}_{i\mathrm{d}} \quad \text{(S34)}$$

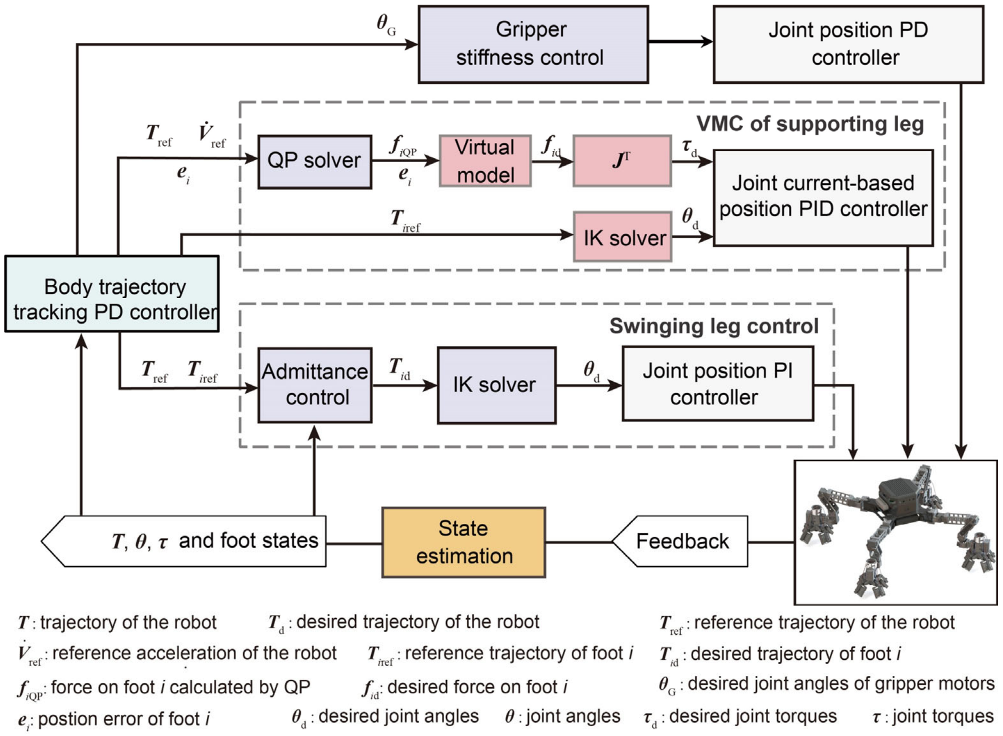

Figure S16. Control architecture of VMC. The different part from the proposed Q-WBC is the supporting leg control loop.

S1.8. Gripper Stiffness Control

In contrast to previous work, which relied solely on the position control mode of the servo motor to allow the winch to rotate to a specified angle, this study introduces a stiffness control method. Given that the elastic components of the SPASAS gripper (e.g., cables, springs) are primarily arranged in series, stiffness control is particularly suitable for gripper operations. The servo motor utilizes PD control to pull the attachment cable, thus driving the fingers to grip and attach. When the motor reaches the desired drive torque threshold, it maintains the servo joint variable constant; however, if the drive torque exceeds the established threshold, the joint variable is reduced to release a small segment of the attachment cable. This strategy prevents servo overload or overheating and reduces unnecessary energy consumption. Conversely, if the

drive torque is insufficient, the motor continues to rotate and tighten the attachment cable to prevent attachment failure due to inadequate drive force.

The control method for the gripper, as shown in Figure S17A. The current torque error e_τ can be obtained from the desired drive torque τ_d and the actual drive torque τ, which is derived from the servo current sensor's feedback current i using the motor current coefficient k_t. Then, e_τ is divided by the transmission system’s equivalent stiffness K_{equ} and scaled by the proportional coefficient $k_{P\tau}$ to determine the desired position θ_d of the output. Trapezoidal velocity and acceleration profiles are used to calculate the position curve $\theta_d(t)$ for the control period, enabling the servo's internal low-level controller to perform PD control of the output position, thereby tracking the desired gripping output torque.

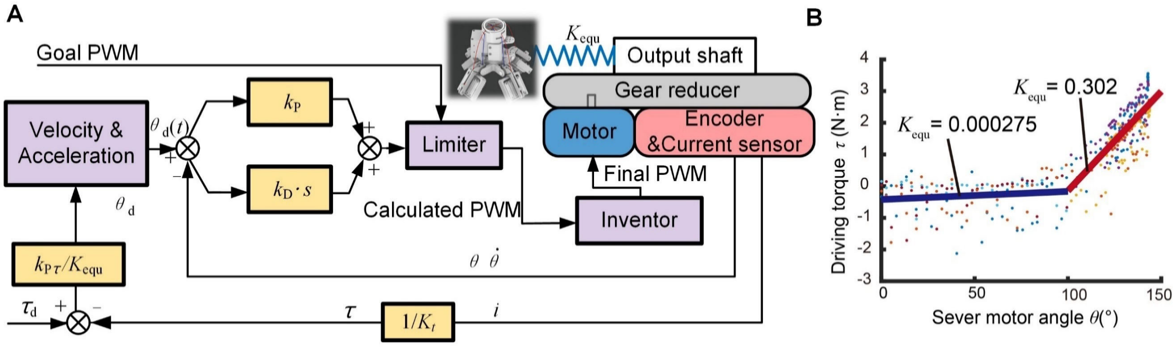

Figure S17. The control method of SPASAS gripper. A) Control flow diagram. **B)** Stiffness characteristic of the gripper.

To effectively implement stiffness control for gripper attachment, recognizing the stiffness characteristics is essential. The gripping process can be categorized into two phases: before and after the fingers make contact with the attachment surface. The equivalent stiffness of the system varies substantially between these stages, necessitating distinct equivalent stiffness curves for each phase. Considering the position as the independent variable and the drive torque as the dependent variable, equivalent stiffness K_{equ} is determined through linear fitting. The position-torque curves indicate a significantly lower K_{equ} in the first phase and a higher K_{equ} in the second phase, as shown in Figure S17B. Consequently, a high gain is chosen in the first phase to increase motion speed without load, while a low gain is used in the second phase to enhance the compliance of attachment. After attachment, the proportional coefficient k_P in the underlying servo controller is increased to improve system response speed. Furthermore, real-time calculation of stiffness also serves as a diagnostic tool to assess whether the attachment is successful. If the measured stiffness value is significantly lower than K_{equ}, it indicates an unsuccessful attachment, necessitating reattachment.

S1.9. Foundation of Mathematics

S1.9.1. PoE Formula

For a given revolute joint, denote that the joint variable is $\theta \in \mathbb{R}$, the unit angular twist is $\boldsymbol{\omega} = [\omega_1 \quad \omega_2 \quad \omega_3]^T \in \mathbb{R}^3$, and the position vector of the joint is $\boldsymbol{r} = [r_1 \quad r_2 \quad r_3]^T \in \mathbb{R}^3$. The skew-symmetric matrix of $\boldsymbol{\omega}$ can be written as

$$\hat{\boldsymbol{\omega}} = \begin{bmatrix} 0 & -\omega_3 & \omega_2 \\ \omega_3 & 0 & -\omega_1 \\ -\omega_2 & \omega_1 & 0 \end{bmatrix} \tag{S35}$$

The PoE formula of the joint can be represented as

$$e^{\theta\hat{\xi}} = \exp\begin{bmatrix} \exp(\theta\hat{\omega}) & \boldsymbol{b} \\ \mathbf{0} & 1 \end{bmatrix} \tag{S36}$$

where

$$\exp(\theta\hat{\omega}) = \mathbf{I}_{3\times3} + \sin\theta\hat{\omega} + (1-\cos\theta)\hat{\omega}^2 \tag{S37}$$

$$\boldsymbol{b} = \left(\theta\mathbf{I}_{3\times3} + (1-\cos\theta)\hat{\omega} + (\theta-\sin\theta)\hat{\omega}^2\right)(\boldsymbol{r}\times\boldsymbol{\omega}) \tag{S38}$$

S1.10.2. Adjoint representation

For the given matrix $\boldsymbol{T} \in SE(3)$, it can be written as

$$\boldsymbol{T} = \begin{bmatrix} \boldsymbol{R} & \boldsymbol{t} \\ \mathbf{0} & 1 \end{bmatrix}_{4\times4} \tag{S39}$$

where $\boldsymbol{R} \in \mathbb{R}^{3\times3}$, and $\boldsymbol{t} \in \mathbb{R}^3$. Its adjoint representation can be represented as

$$\mathrm{Ad}(\boldsymbol{T}) = \begin{bmatrix} \boldsymbol{R} & \boldsymbol{0} \\ \boldsymbol{R}\hat{\boldsymbol{t}} & \boldsymbol{R} \end{bmatrix} \tag{S40}$$

For the matrix $\boldsymbol{V} = \begin{bmatrix} \boldsymbol{\omega} \\ \boldsymbol{v} \end{bmatrix} \in \mathbb{R}^6$, where its $\boldsymbol{\omega} \in \mathbb{R}^3$ and $\boldsymbol{v} \in \mathbb{R}^3$, its adjoint represent is denoted as

$$\mathrm{ad}(\boldsymbol{V}) = \begin{bmatrix} \hat{\boldsymbol{\omega}} & \mathbf{0} \\ \hat{\boldsymbol{v}} & \hat{\boldsymbol{\omega}} \end{bmatrix} \tag{S41}$$

S2. Gripper Load Capacity Test

Here, attachment force tests were conducted on basalt surfaces as shown in Figure S18. These rock surfaces are very rough and porous, making them feasiblely attached to by the spiny gripper. The tests used 10 samples, as illustrated in Figure S18A and B. Normal force tests were performed on samples 1 to 5, while the shear force tests were carried out the on samples 6 to 10 of the climbing test platform. During the tests, four different orientations were randomly selected for grasping each rock. Subsequently, weights were suspended from the rock or the SPASAS gripper for loading. The total weight loaded served as the measure of the normal or shear attachment force produced by the gripper, as depicted in Figure S18C and D. It is important to note that due to safety reasons, the ultimate attachment forces were not measured in some groups.

Figure S18C shows the results of the normal force tests conducted on samples 1 to 5, while Figure S18D shows the results of the shear force tests on samples 6 to 10. The test results indicate that the SPASAS gripper can generate a minimum shear adhesion force of 49.0 N and a normal attachment force of 43.7 N on the tested basalt surfaces. The maximum shear adhesion force produced by the gripper is 61.3 N, and the maximum normal adhesion force is 76.9 N. The weight of the gripper is 3.82 N, and the robot's weight is 47.04 N. The test results demonstrate that the gripper can provide sufficient attachment force for medium and small-sized climbing robots to perform climbing movements.

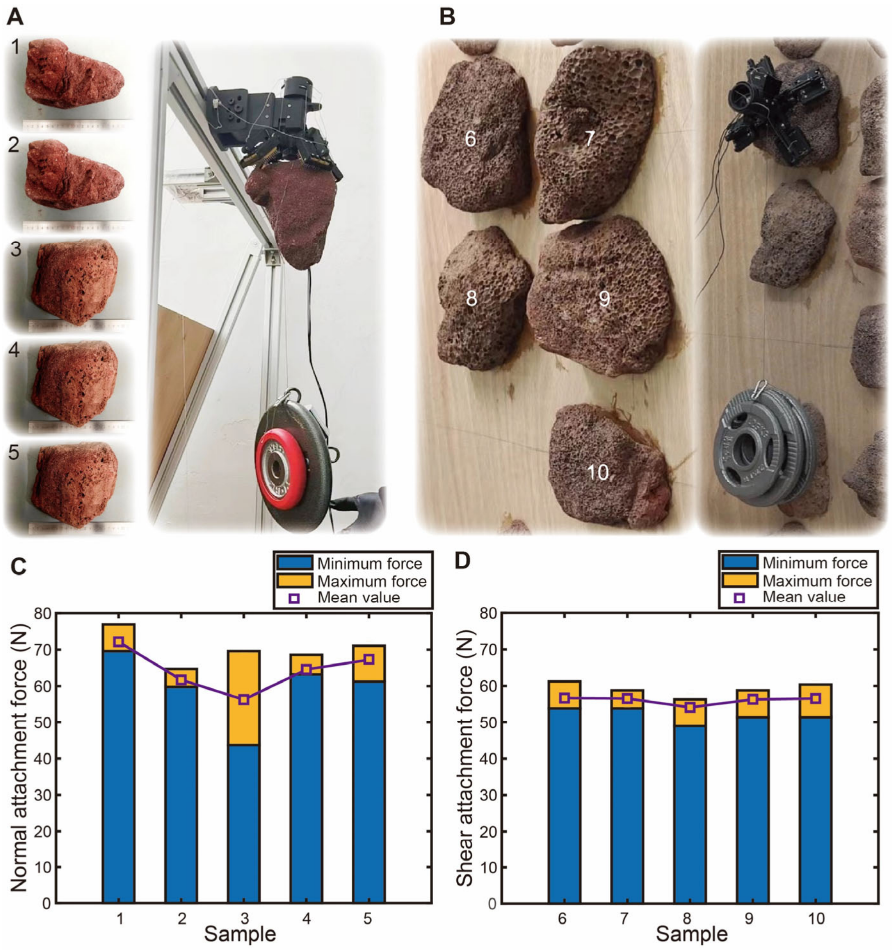

Figure S18. Results of the gripper load test. **A)** Samples and method for normal attachment force test. **B)** Samples and method for shear attachment force test. **C)** Normal attachment force. **D)** Shear attachment force.

S2. Discussion

S2.1. Feasibility of Spine Mechanism in Extreme Terrain

Previous studies assumed the tip of the spine to be a spherical surface [9]. A smaller tip radius allows the spine to generate better mechanical interlock with the tiny asperities of the attached surface. However, concentration of contact force on a small number of spines increases the risk of deformation and bending of the spine tips. Therefore, when dealing with rough terrain at the millimeter and centimeter scale, it is recommended to choose a larger tip radius to prevent bending. It should be noted that deformation of the spine tip is unavoidable during prolonged use. Furthermore, we consider that the deformation of the spine tip had limit impact on the gripper's adhesion force or success rate. This can be attributed to the roughness of the target surface, which is significantly larger than the spine tip. Even after deformation, the radius of the spine tip remains much smaller than the roughness of the surface. This finding highlights the long-term durability of the spine mechanism in facilitating the attachment and movement of unmanned systems.

S2.2. Visual Sensing Scheme Limitations

The depth and binocular cameras were not utilized for terrain recognition and climbing in the experiments due to the artificial and predefined nature of the terrain. The low height of the fuselage may obstruct the view of the cameras by the limbs. To address this, a cloud terrace module should be added to the camera in future research to expand its field of view. However,

relying solely on the camera mounted on top of the fuselage makes it challenging to obtain accurate foothold information. An improvement would be to add small visual or infrared sensors to each of the robot's limbs, enabling the robot to assess the suitability of the local terrain for attachment.

S2.3. Gripper Attachment Rigidity during Climbing

It is assumed in the robot's kinematics and dynamics modeling that once an attachment is formed between the gripper and the rock surface, the position of the attachment device relative to the attached surface will not change. However, during climbing movements, the ends of the robot's limbs are not completely fixed to the target surface. The elastic element in the attachment device may deform when the attachment force varies significantly, and the spines may experience slight slippage on the rock surface. Additionally, the attachment force may dislodge asperities on the rock, and the spine tips may deform due to stress concentrations. These factors can result in relative motions between the gripper and the attached surface, which can both disrupt the robot's stability and introduce errors in state estimation. Therefore, to obtain a more precise measurement of the gripper's position, an IMU can be integrated into the gripper. By incorporating IMU feedback at the end of the limb during state estimation, the observed value can be brought closer to the actual value.

S2.4. Hexapod Robot versus Quadruped Robot

Compared to hexapod robots, quadruped robots are gradually becoming more popular in research due to their simpler structure, higher mobility efficiency, and speed. However, when faced with challenging terrain, hexapod robots clearly exhibit higher reliability. It has been demonstrated and discussed that the transition of the limbs from the attached state to the swinging state is the most vulnerable moment for the robot to become dislodged. To ensure the reliability of climbing in this robot, the stability of each attachment device must be ensured first and foremost. This is a disadvantage of quadruped robots, as their mechanisms and structures do not provide redundancy in terms of reliability for potential risks. If a hexapod configuration is used for the climbing robot, the design, motion planning, and control become more complex, the number of constraints imposed by the environment on the limbs increases, and the real-time performance of kinematics and dynamics solving is affected. However, when a limb is in the switching state, the magnitude of the change in the attachment wrench of the other limbs should be smaller than that of a quadruped robot, allowing the robot to be subjected to less disturbance. Having more limbs may also provide redundancy for the climbing motion, ensuring that even if any of the limbs or grippers malfunction or fail, the entire system remains operational. Nevertheless, the discussion about hexapod climbing robots is currently based on assumptions and inferences, and further research and comparisons of such prototypes are required.

Supplementary References

[1] J. He, F. Gao, *Chin. J. Mech. Eng.* **2020**, 33, 1.
[2] P. J. Zi, K. Xu, Y. B. Tian, X. L. Ding, *Mech. Mach. Theory* **2023**, 181, 105168.
[3] Y. Gao, W. Wei, X. Wang, Y. Li, D. Wang, Q. Yu, *Appl. Intell* **2021**, 1.
[4] J. Dai, *Screw algebra and kinematic approaches for mechanisms and robotics*, Springer, London, **2014**.
[5] A. Parness, N. Abcouwer, C. Fuller, N. Wiltsie, J. Nash, B. Kennedy, presented at *2017 IEEE Int. Conf.on Robotics and Automation* **2017**.
[6] R. W. Brockett, Berlin, Heidelberg, **1984**.
[7] a)M. Focchi, A. Del Prete, I. Havoutis, R. Featherstone, D. G. Caldwell, C. Semini, *Auton. Robot.* **2017**, 41, 259; b)J. Di Carlo, P. M. Wensing, B. Katz, G. Bledt, S. Kim, presented at *2018 IEEE/RSJ Int. Conf. on Intelligent Robots and Systems* **2018**.
[8] a)F. Bullo, R. M. Murray, presented at *Proc. of the European Control Conference*, Rome, Italy, June 1995, **1995**; b)J. Park, W.-K. Chung, *IEEE Trans. Rob.* **2005**, 21, 850; c)Y. Yu, X. Ding, J. J. Zhu, *J. Frankl. Inst.-Eng. Appl. Math.* **2013**, 350, 2044.
[9] a)Z. Dai, S. N. Gorb, U. Schwarz, *J. Exp. Biol.* **2002**, 205, 2479; b)A. T. Asbeck, S. Kim, M. R. Cutkosky, W. R. Provancher, M. Lanzetta, *Int. J. Rob. Res.* **2006**, 25, 1165.

Supplementary Movies

Movie S1: this movie is the video abstract of the whole work. It briefly introduced the background of the study, the design and control method of the robot, the experiment results, and the primary contribution.

Movie S2: the movie shows the attachment and detachment process, and the load capacity of the developed gripper.

Movie S3: this movie records the climbing locomotion of MARCBot in a vertical rock surface.

Movie S4: this movie records the climbing locomotion of MARCBot in a 70-degree incline rock surface.

Movie S5: this movie records MARCBot using the wave climbing mode to sacle a vertical rock surface.

Movie S6: this movie records the trotting locomotion of MARCBot in complex terrains including gravel terrain, grass, muddy terrain, and stone path.
